# Breaking the grain size limit of glass-crystallized transparent nanoceramics via multi-phase interface locking

**DOI:** 10.1038/s41467-026-76601-5

**Published:** 2026-08-15

**Authors:** Wenlong Xu, Yongchang Guo, Haohan Fan, Yuanfeng Liu, Zhibiao Ma, Linghan Bai, Ruyu Fan, Tiannan Zhang, Guoguo Zhang, Yuhang Chen, Shijiao Zhang, Chuanbao Liu, Binghui Ge, Jingbo Sun, Yanhao Dong, Jianqiang Li

**Affiliations:** 1 School of Materials Science and Engineering, University of Science and Technology Beijing, Beijing, China; 2 University of Chinese Academy of Sciences, Beijing, China; 3 State Key Laboratory of Opto-Electronic Information Acquisition and Protection Technology, Leibniz International Joint Research Center of Materials Sciences of Anhui Province, Institutes of Physical Science and Information Technology, Anhui University, Hefei, China; 4 State Key Lab of New Ceramic Materials, School of Materials Science and Engineering, Tsinghua University, Beijing, China; 5 Beijing Key Laboratory for Advanced Powder Metallurgy and Particulate Materials, University of Science and Technology Beijing, Beijing, China

**Keywords:** Ceramics, Glasses

## Abstract

Nanoceramics from sintering routes are challenging to process due to difficulties in deagglomerating nanopowders, suppressing grain growth, and eliminating macroscopic defects. Glass crystallization offers new possibilities by first densifying materials to optical transparency in amorphous form and then fully crystallizing into ceramic form. However, refining the grain size within the nanocrystalline regime is again challenging, due to rapid grain growth under large crystallization driving force and nucleation-controlled kinetics. Here we tackle the problem by decomposing the homogeneous glass precursors into ternary phase composite ceramics, thus coupling the coarsening kinetics to slow cation diffusion required for phase partitioning and hetero-phase boundary motion required for grain growth. The design allows precise adjustment of the evolving microstructure and yields a fine grain size down to ~16 nm in fully crystallized ceramics. We demonstrate that the obtained nanoceramics can deliver high optical transparency, despite the different refractive indices of the cubic La(Al_1/3_Ti_2/3_)O_3_, Y_2_(Zr_0.6_Ti_0.4_)_2_O_7_ and transitional Al_2_O_3_ phases, and improved mechanical properties. Our work unlocks the hidden opportunities of designing and producing phase-complex nanoceramics in high-dimensional compositional space beyond the traditional phase-pure solubility constraint.

## Abstract (web summary)

Multi-phase interface locking enables glass-crystallized transparent nanoceramics to retain sub-20 nm grains even with three coexisting crystalline phases, achieving high transparency, a high-index/Abbe combination, and enhanced mechanical properties

## Introduction

Transparent ceramics are critical in high-value applications such as medical diagnostics, high-power lasers, precision sensors, and extreme-environment technologies, owing to their exceptional opto-thermo-mechanical performance^1–4^. As polycrystalline materials, the optical transmittance is governed by light scattering from grain boundaries and residual porosity^5,6^. Consequently, according to Rayleigh scattering theory^7^, suppressing the grain size below 1/5–1/10 of the incident wavelength while minimizing residual porosity is beneficial to improve the optical transmittance. In addition, grain size refinement brings mechanical advantages such as improved hardness and strength as predicted by the Hall–Petch relationship. These highlight the significance of developing nanocrystalline transparent ceramics.

In the conventional route, processing of transparent ceramics relies on powder techniques and pressure-assisted high-temperature sintering for densification and complete pore elimination^8–11^. One of the most challenging issues is to decouple the kinetics of sintering and grain growth, both driven by the same capillarity force to minimize interfacial excess energy. As a result, nanoceramics in general^12^, particularly nanocrystalline transparent ceramics, are extremely difficult to prepare. On the other hand, the glass crystallization route of transparent ceramics has been developed by crystallizing the dense glass sample into polycrystalline ceramics above the glass transition temperature^7,13–16^. In this way, the kinetics of sintering and grain growth are naturally decoupled into two different steps. Nevertheless, the crystallization step by itself is often nucleation-controlled and under a large chemical driving force between the undercooled glass phase and the thermodynamically stable crystalline phase. As a result, nanoceramics from this route are rarely obtained, even in the case of GPa-level pressure-assisted crystallization^9^. Complete crystallization while retaining <100 nm grain size remains a challenge to be resolved.

We believe new opportunities exist in kinetic control of the grain growth during the crystallization step via interface pinning. This is because grain growth in single-phase materials takes place by grain-boundary migration of atomic shuffling and disconnection movement, while that in multiphase materials must be coupled to longer-range atomic diffusion of more atomic fluxes. It is for the same reason why multiphase composites can efficiently suppress both static and dynamic grain growth under harsh thermos-mechanical conditions, as is the case in high-strain-rate ceramic superplasticity^17^. To design multiphase nanocrystalline transparent ceramics prepared by the glass crystallization route, several factors also need to be taken into account. First, nanoscale grain sizes can significantly suppress grain-boundary scattering and mitigate birefringence effects of visible light^18^. Second, preferential precipitation of cubic crystalline phases or non-cubic phases with weak birefringence is required to maintain optical isotropy, thereby suppressing transmission losses from birefringence-induced light scattering. Third, during the crystallization of glass, a sufficiently small volume contraction is required to prevent crack formation and achieve a sufficiently dense structure. This implies that the density difference between the glass phase and the crystalline phase should be minimized. Alternatively, the presence of certain analogous transition domains, such as transition Al_2_O_3_ domains, can effectively release the stresses induced by this contraction^14,19,20^. Compositions with high nucleation rate and low growth rate can also be selected to obtain nano-sized grains^21^.

The glass crystallization route imposes stringent requirements on the compositions of precursor glasses, rendering this method inherently unscalable. Up to now, glass precursors utilized in this route have been predominantly limited to binary and ternary oxide systems^22–26^, which frequently yield single-phase or dual-phase structures. Although multi-principal element glasses (MPEGs) of R_2_O_3_-Y_2_O_3_-TiO_2_-ZrO_2_-Al_2_O_3_ (R = La, Sm, Gd, referred to as RYTZA)^27^ with enhanced mechanical properties have recently been developed, their conversion into transparent ceramics via glass crystallization has not yet been demonstrated. These MPEGs thus serve as a promising platform for exploring such transparent nanoceramics.

This study systematically investigates the crystallization behavior of MPEGs. Under mild, low-temperature, pressure-free processing conditions, we successfully synthesized perovskite-based transparent nanoceramics with an average grain size of ~16 nm (determined by the linear intercept method). The nanoceramics retain excellent optical transmittance while exhibiting a significant enhancement of over 50% in mechanical properties. These advancements expand the application potential of MPEGs in extreme service environments. Furthermore, the nanocrystalline perovskite-based ceramics demonstrate exceptional potential for laser lighting and high-resolution displays. Crucially, this work establishes a multiphase interface locking strategy for nanocrystalline transparent ceramics. Future exploration of crystallization in more complex glass systems (e.g., high-performance MPEGs^28–30^) could enable the development of functionally diversified transparent ceramics.

## Results

### Phase evolution and suppressed grain growth

**Fig. 1:**
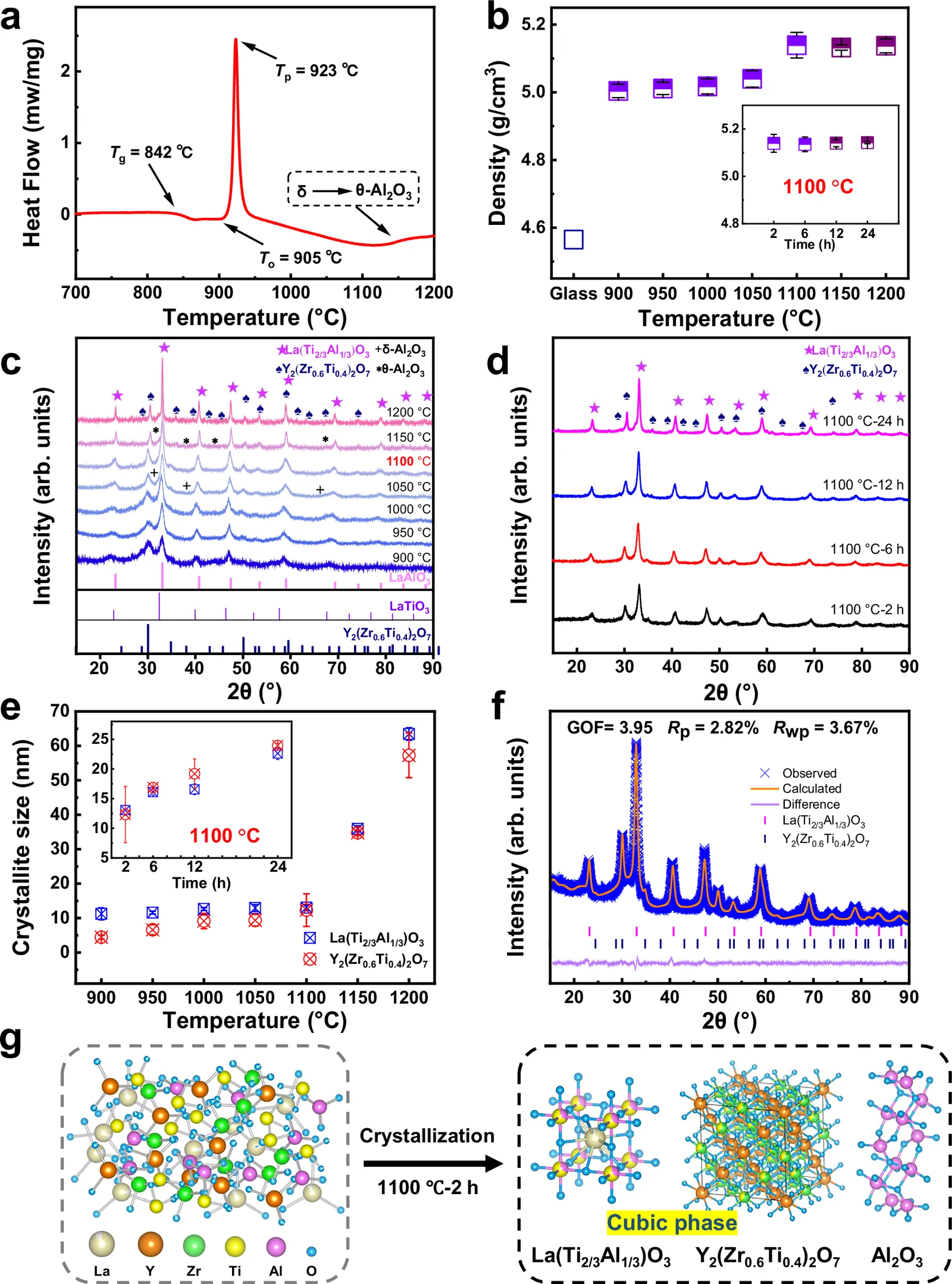
Crystallization kinetics of LYTZA glass. **a** DSC curve. **b** Density of the samples crystallized at 900–1200 °C for 2 h (7200 s). Data are presented as mean values, and error bars represent standard deviation from *n* = 10 technical replicates (independent measurements on the same sample). The inset shows the density of samples crystallized from LYTZA glass at 1100 °C for 2–24 h. **c**, **d** X-ray powder diffraction (XRD) patterns of samples crystallized from LYTZA glass at 900–1200 °C for 2 h (**c**) and at 1100 °C for 2–24 h (**d**). Measurements were performed with Cu Kα radiation (*λ* = 1.54059 Å). **e** Crystallite sizes calculated from XRD data of the samples crystallized at 900–1200 °C for 2 h (the inset shows crystallite sizes of samples crystallized at 1100 °C for 2–24 h). Data are presented as mean values, and error bars represent standard deviation derived from three diffraction peaks for La(Al_1/3_Ti_2/3_)O_3_ and two diffraction peaks for Y_2_(Zr_0.6_Ti_0.4_)_2_O_7_. **f** XRD patterns of the multiphase nanocrystalline ceramics crystallized at 1100 °C for 2 h. **g** Schematic diagram of glass crystallization structure transformation. Source data are provided as a Source Data file.

The multi-principal element LYTZA glass exhibits complex crystallization behaviors arising from its non-equilibrium stoichiometry. Phase transitions were analyzed using differential scanning calorimetry (DSC), as shown in Fig. 1a. The glass transition temperature (*T*_g_) was identified at 842 °C, followed by the onset of crystallization (*T*_onset_) at 905 °C and the peak crystallization temperature (*T*_p_) at 923 °C. A weak exothermic event observed between 1100 °C and 1200 °C (*T*_onset_ = 1120 °C) corresponds to the δ- to θ-Al_2_O_3_ transition. This assignment is corroborated by X-ray powder diffraction (XRD) analysis, which reveals the emergence of δ-Al_2_O_3_ at 1050 °C and θ-Al_2_O_3_ at 1150 °C, consistent with the DSC profile. These results align with the Al_2_O_3_ phase transitions observed during the crystallization of transparent yttrium aluminum garnet-based nanoceramics in previous research^14,19^. Based on these results, the LYTZA glass precursor was processed under thermal conditions spanning 900–1200 °C with a 2 h isothermal hold to achieve complete crystallization and fabricate transparent ceramics.

Density measurements provide indirect evidence for the completion of crystallization and phase transition. As shown in Fig. 1b, the density stabilized at 5.139 g cm^−3^ after crystallization at 1100 °C for 2 h. This represents an increase of 12.57% relative to the glass precursor (4.565 g cm^−3^), 2.7% relative to the sample crystallized at 900 °C for 2 h (5.004 g cm^−3^), and only about 0.14% difference relative to the samples crystallized at 1150 °C (5.132 g cm^−3^) and 1200 °C (5.137 g cm^−3^) for 2 h, with no significant change (within 0.07%) observed upon further extending the holding time (5.135–5.142 g cm^−3^). Based on the nominal composition, the theoretical density of the ceramic was calculated to be 5.142 g cm^−3^, which is in good agreement with the measured density of 5.139 g cm^−3^. This evolution in density, together with the consistency between the measured and theoretical values, provides further evidence that the crystallization process has reached completion.

XRD analysis (Fig. 1c) reveals the phase evolution during crystallization at 900–1200 °C for 2 h. The diffraction peaks correspond to LaTiO_3_ (PDF Card No.75-0267), LaAlO_3_ (PDF Card No.70-4127), and Y_2_(Zr_0.6_Ti_0.4_)_2_O_7_ (PDF Card No.85-1585). The perovskite peak positions between LaTiO_3_ and LaAlO_3_ suggest Ti/Al co-occupancy at the B-site (aligned with the Scanning transmission electron microscopy (STEM)–Energy-dispersive X-ray spectroscopy (EDS) results). At 900 °C, these phases coexist with amorphous features (broad/missing diffraction peaks), which diminish as temperature rises. Complete crystallization (sharp peaks, no amorphous signals) is achieved at 1100 °C, corresponding to the structural transformation schematically represented in Fig. 1g. This is corroborated by Fig. 1d, which shows no additional phases emerging when the crystallization time is extended from 2 to 24 h at 1100 °C, confirming that the transformation is complete within 2 h. The microstructural diversity inherent to this multi-principal element system generates abundant, efficient nucleation sites^21^, facilitating the simultaneous precipitation of multiple crystalline phases. XRD analysis further reveals that both La(Al_1/3_Ti_2/3_)O_3_ and Y_2_(Zr_0.6_Ti_0.4_)_2_O_7_ phases adopt cubic symmetry^31,32^, a feature beneficial to optical transparency.

**Fig. 2:**
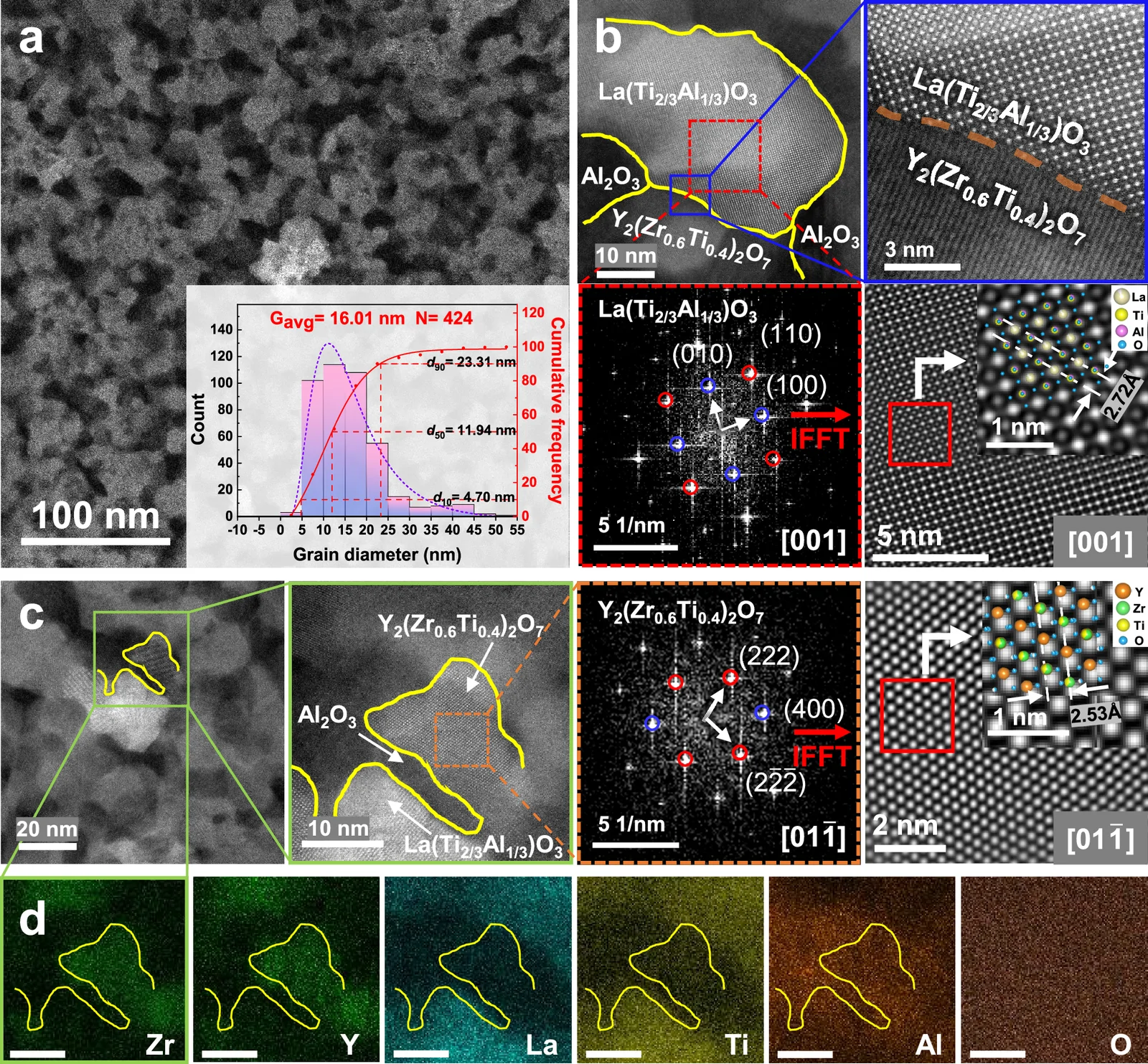
High-angle annular dark-field (HAADF)–Scanning transmission electron microscopy (STEM) observations and Energy-dispersive X-ray spectroscopy (EDS) mapping results of the multiphase nanocrystalline ceramic crystallized from LYTZA glass at 1100 °C for 2 h. **a** HAADF–STEM micrograph with the corresponding grain size distribution shown in the inset. **b**, **c** Higher-magnification HAADF–STEM images revealing three distinct phases: the light phase is identified as La(Al_1/3_Ti_2/3_)O_3_, the gray phase as Y_2_(Zr_0.6_Ti_0.4_)_2_O_7_, and the dark phase as Al_2_O_3_. The corresponding Fast Fourier Transform (FFT) patterns and inverse FFT images are provided for the La(Al_1/3_Ti_2/3_)O_3_ and Y_2_(Zr_0.6_Ti_0.4_)_2_O_7_ phases. Insets display a higher-magnification view along with simulated models generated in Vesta using structural parameters derived from XRD refinement, projected along the [001] and [01$\bar{1}$] zone axes for La(Al_1/3_Ti_2/3_)O_3_ and Y_2_(Zr_0.6_Ti_0.4_)_2_O_7_, respectively. **d** EDS elemental maps of the region marked by the green box in (**c**), with a scale bar of 10 nm (1 × 10^−8 ^m). All experiments were independently repeated three times with similar results; representative images are shown. Source data are provided as a Source Data file.

Grain sizes, analyzed using the Scherrer equation^33^ after correcting for instrumental broadening (via the full width at half maximum, FWHM), reveal a gradual increase with crystallization temperature. The analysis is supported by data presented in Fig. 1e, Supplementary Fig. 1, Supplementary Table 1, and Supplementary Eq. (1). At 1100 °C, La(Al_1/3_Ti_2/3_)O_3_ and Y_2_(Zr_0.6_Ti_0.4_)_2_O_7_ grains reach 13.1 ± 0.8 nm and 12.3 ± 4.7 nm, respectively, consistent with STEM image (Fig. 2a). Above 1100 °C, grain size increases two–threefold, primarily caused by the disappearance of the amorphous network (reducing growth barriers) and elevated sintering temperature (lowering grain-boundary energy and enhancing mobility)^34,35^. Time-dependent experiments further elucidate the grain growth kinetics. The sample crystallized at 1100 °C undergoes further grain growth after 12 h and yields ~23 nm grain size at 24 h, which is still smaller than those obtained from high-temperature short-term processes (1150 °C, 2 h: ~35 nm). This comparison underscores that temperature exerts a more dominant influence than time on grain growth in this system.

Quantitative phase analysis was performed via Rietveld refinement of the XRD pattern for the sample crystallized at 1100 °C for 2 h (Fig. 1f). Owing to the weak diffraction intensity of the Al_2_O_3_ phase, which could compromise refinement accuracy, only the perovskite and the Y_2_(Zr_0.6_Ti_0.4_)_2_O_7_ phases were selected for refinement. The refinement results indicate a 2:1 ratio of Ti to Al at the B-site of the perovskite phase, in agreement with the subsequent STEM–EDS analysis. The refined molar ratio of La(Al_1/3_Ti_2/3_)O_3_ to Y_2_(Zr_0.6_Ti_0.4_)_2_O_7_ is 85.3% to 14.7%, closely matching the nominal composition ratio of 88.6% to 11.4%. The refined unit cell parameter of La(Al_1/3_Ti_2/3_)O_3_ (*a* = 3.83546 Å) lies between those of LaAlO_3_ (ICSD 153836; a = 3.8278 Å) and LaTiO_3_ (ICSD 28908; *a* = 3.92 Å), reflecting the B-site Al/Ti co-occupancy effect. For Y_2_(Zr_0.6_Ti_0.4_)_2_O_7_ (ICSD 66875), the refined *a* = 10.2711 Å aligns closely with its reference value (10.291 Å). These results collectively validate the phase composition and structural consistency of the crystallized product.

### Microstructure of multiphase nanocrystalline ceramics

The HAADF–STEM image (Fig. 2) and the selected area electron diffraction (SAED) pattern (Supplementary Fig. 2) of the multiphase nanocrystalline ceramic crystallized at 1100 °C for 2 h both confirm extensive crystallization, with the SAED pattern revealing sharp reflections from three distinct phases and no detectable amorphous regions. The sample is also fully densified, free of pores and microcracks. Quantitative analysis of the grain size distribution via the linear intercept method (Fig. 2a, based on 424 measurements) reveals an average grain size of ~16.0 nm. This STEM‑derived value is consistent with XRD analysis, which yields grain size estimates consistently below 20 nm. The Scherrer equation provides a convenient estimate of the coherent diffraction domain size, but it assumes spherical, strain‑free crystallites and may be influenced by microstrain and instrumental broadening^36^, despite correction for instrumental broadening. These values should therefore be interpreted as volume‑weighted averages rather than absolute measures of physical grain dimensions^37^. By contrast, STEM analysis offers direct visualization of individual grains and enables statistical sampling across multiple regions. The close agreement between the XRD‑derived values and the STEM statistical analysis cross‑validates the two methods and confirms the reliability of the reported grain size range.

The grain size achieved via a pressureless densification route is remarkably smaller than those obtained from conventional sintering and previous glass crystallization techniques (Supplementary Table 3). Specifically, the grain sizes from conventional sintering are limited by initial powder size and coarsening during densification, and the best practices of pressureless sintering are 30–60 nm^38,39^. The grain sizes from previous glass crystallization techniques are limited by a few nucleation sites and rapid coarsening before full crystallization, and the best practices are above 100 nm. To overcome this limitation, advanced synthesis strategies have been explored. Irifune et al.^10^ achieved a grain size control of 30–50 nm by crystallization under ultra-high-pressure conditions. For instance, in our group’s previous work, by introducing Al_2_O_3_ as the second phase to restrict the grain growth, the minimum grain size of about 30 nm was obtained (Y_3_Al_5_O_12_-Al_2_O_3_ nanoceramics crystallized at 1100 °C for 2 h with a grain size of 30 nm^19^, the grain size of Lu_3_Al_5_O_12_-Al_2_O_3_ nanoceramics is 65.7 ± 2.3 nm after crystallization at 1100 °C for 2 h^20^). Notably, fully crystallized ceramics with sub-20 nm grains have rarely been achieved (Supplementary Table 3).

In this work, the remarkable reduction in grain size can be attributed to two synergistic effects. First, the multi‑component nature of the initial glass precursor creates a complex chemical environment that not only slows cation diffusion required for phase partitioning, but also provides abundant nucleation sites due to lattice distortions. This enables the simultaneous precipitation of three distinct crystalline phases from the homogeneous glass matrix. The rapid and concurrent formation of multiple phases establishes, from the very beginning of crystallization, a dense network of heterophase boundaries that sets the stage for subsequent growth inhibition. Second, once this multiphase nanostructure is formed, grain growth becomes kinetically constrained by a more fundamental mechanism. For a grain of any given phase to grow, atoms must undergo long‑range diffusion from source regions to sink regions of the same phase, a process that requires traversing multiple grains of dissimilar compositions. This stands in stark contrast to grain growth in single‑phase materials, which proceeds via relatively rapid local mechanisms such as atomic shuffling and disconnection movement at grain boundaries. The sluggish growth kinetics arising from this diffusion‑coupled constraint effectively suppresses coarsening, locking in the nanoscale multiphase structure.

It is instructive to compare this mechanism with our previous two‑phase YAG–Al_2_O_3_ system^19^, where Al_2_O_3_ acted primarily as a physical barrier imposing self‑limited grain growth to ~30 nm. While effective, that mechanism relied on passive obstruction. In the present three‑phase system, the coupling of grain growth to compositionally constrained, long‑range diffusion represents a more fundamental kinetic limitation—pushing the grain size below 20 nm. This slow growth kinetics not only suppresses dynamic grain coarsening but also accounts for the material’s ability to maintain high transparency despite complex multiphase coexistence, as phase boundaries at this scale become negligible scattering centers. Furthermore, such kinetically arrested microstructures are known to enable high‑strain‑rate superplasticity at elevated temperatures, highlighting additional functional potential of this interface‑locking strategy.

To better visualize the three-phase microstructure, regions with distinct phase contrast and slightly coarser grains were selected for imaging. Higher-magnification HAADF–STEM images in Fig. 2b, c reveal a uniformly distributed three-phase microstructure comprising bright (La(Al_1/3_Ti_2/3_)O_3_, Z̅ = 20), gray (Y_2_(Zr_0.6_Ti_0.4_)_2_O_7_, Z̅ = 18.15), and dark (Al_2_O_3_, Z̅ = 10) contrast phases, with no observed phase aggregation. Further magnified images resolve the atomic-scale interface between the La(Al_1/3_Ti_2/3_)O_3_ and Y_2_(Zr_0.6_Ti_0.4_)_2_O_7_ phases, revealing a sharp, defect-free grain boundary only a few atomic layers thick, devoid of pores or impurities. Fast Fourier Transform (FFT) analysis was used to identify both major phases and determine their crystallographic orientations. For La(Al_1/3_Ti_2/3_)O_3_ viewed along [001], the measured (110) d-spacing (2.72 Å) lies between those of LaTiO_3_ (2.7719 Å) and LaAlO_3_ (2.7073 Å), consistent with B-site Al/Ti co-occupancy. For Y_2_(Zr_0.6_Ti_0.4_)_2_O_7_ viewed along [01$\bar{1}$], the (400) d-spacing (2.53 Å) closely matches the reference value (2.5728 Å).

STEM–EDS element mapping (Fig. 2d) reveals co-localization of Zr and Y, confirming the Y_2_(Zr_0.6_Ti_0.4_)_2_O_7_ phase. Although Ti content in this phase is low, the mapping clearly distinguishes the three phases: bright La(Al_1/3_Ti_2/3_)O_3_, gray Y_2_(Zr_0.6_Ti_0.4_)_2_O_7_, and dark Al_2_O_3_. This phase assignment is consistent with XRD results. Quantitative analysis of the region within the green box in Fig. 2c (Supplementary Table 2) indicates a La:Ti ratio of ~3:2, matching the refined composition of La(Al_1/3_Ti_2/3_)O_3_. This result suggests that all La_2_O_3_ present in the glass is consumed to form La(Al_1/3_Ti_2/3_)O_3_ nanocrystals, while the excess Al_2_O_3_ in the LYTZA glass results in transition Al_2_O_3_ domains during crystallization.

### Effect of nanocrystalline grains on optical and mechanical properties

**Fig. 3:**
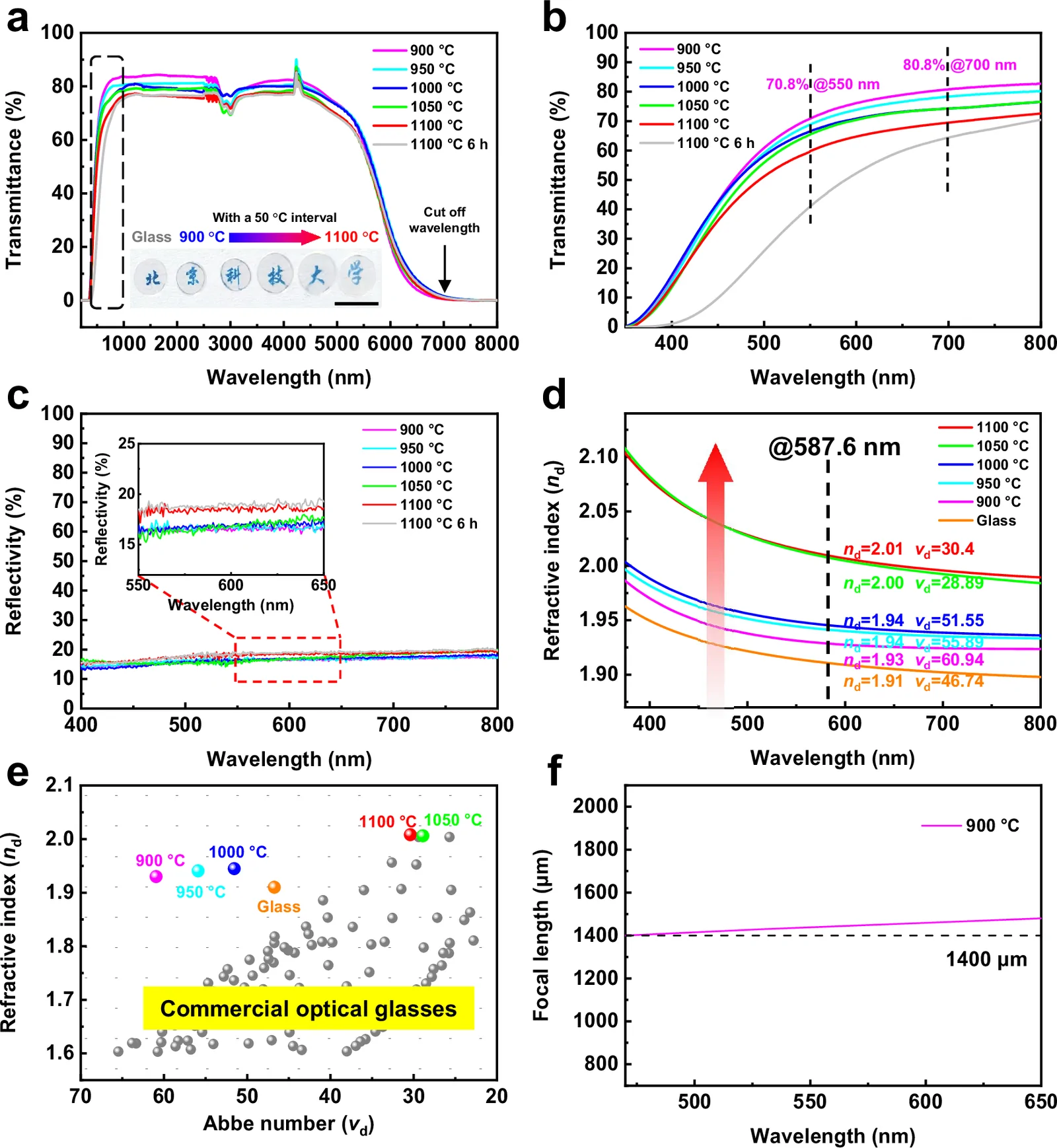
Optical properties of LYTZA glass after crystallization. **a** In-line transmittance spectra of samples with a thickness of 1 mm crystallized from LYTZA glass at 900–1100 °C for 2 h and 1100 °C for 6 h (The inset shows photographs of LYTZA glass samples crystallized at 900–1100 °C for 2 h, placed on a polycarbonate board positioned 2 cm above the background. The scale bar is 5 mm.). **b** Enlarged view of the 350–800 nm spectral region from (**a**), corresponding to the area marked by the black dashed box. **c** Reflectance spectrum in the 400–800 nm range of LYTZA glass crystallized at 900–1100 °C for 2 h and 1100 °C for 6 h. **d** Refractive index (*n*) versus wavelength of samples crystallized at 900–1100 °C for 2 h. **e** Refractive index at 587.6 nm versus Abbe number (*ν*_d_) for the prepared samples compared with commercial optical glasses. The original figure has been enlarged and is available, together with the cited reference, as Supplementary Fig. 5. **f** Focal length of LYTZA glass crystallized at 900 °C for 2 h. Source data are provided as a Source Data file.

Despite the formation of a complex three-phase structure, the crystallized samples exhibit high transmittance across the ultraviolet–visible–near-infrared (UV–Vis–NIR) and mid-infrared (MIR) regions. As shown in Fig. 3a (1.0 mm thickness), the in-line transmittance (T) decreases with increasing crystallization temperature. For the sample crystallized at 900 °C for 2 h, the transmittance at 700 nm and 550 nm is 80.8% and 70.8%, respectively; after crystallization at 1100 °C for 2 h, these values drop to 69.5% and 59.9% (Fig. 3b). Samples become fully opaque after 12–24 h (Supplementary Fig. 3). This reduction correlates with increased reflectance (R) and absorbance (A) at higher temperatures (Fig. 3c and Supplementary Fig. 4a). Extending the crystallization time to 6 h at 1100 °C further elevates absorbance (calculated as A = 1 − T − R, thus including scattering losses), consistent with the observed substantial growth of crystal grains (Fig. 1e).

The coexistence of three crystalline phases without significant loss of transparency represents a striking departure from conventional expectations in ceramic optics, where multiphase interfaces typically act as strong scattering centers. This apparent contradiction is resolved by considering the dominant role of grain size: when grain dimensions are reduced to the deep sub-wavelength scale (<20 nm), the phase boundaries themselves become smaller than the wavelength of visible light, rendering them negligible as scattering sources. The key insight is that transparency is governed by the size of the scattering features relative to the wavelength, not by phase purity per se. Both Apetz and van Bruggen’s transmittance prediction model^18^ (based on Rayleigh–Gans–Debye theory) and Harris et al.’s model^40^ (incorporating reflection, absorption, and Mie scattering) indicate that when the grain size falls below a critical threshold, Rayleigh scattering becomes the dominant mechanism, leading to negligible scattering losses. For green light (*λ* ≈ 550 nm), our achieved grain size of <20 nm corresponds to <*λ*/27—well within the regime where scattering becomes essentially negligible. This size-dominated transparency paradigm explains how multiphase coexistence can be reconciled with high optical performance. Specifically, three synergistic factors underpin the observed transmittance: (1) full densification resulting from a homogeneous glass precursor, eliminating porosity-related scattering; (2) cubic symmetry of both La(Al_1/3_Ti_2/3_)O_3_ and Y_2_(Zr_0.6_Ti_0.4_)_2_O_7_ phases, ensuring optical isotropy and eliminating birefringence; and (3) most critically, the nanocrystalline grain size (<20 nm) that pushes phase boundaries below the scattering threshold. Among these, the grain size effect is uniquely enabling: without it, even fully dense, optically isotropic multiphase ceramics would still suffer from phase-boundary scattering. The nanocrystalline grains effectively “hide” the multiphase interfaces from incident light, allowing the material to behave optically as a homogeneous medium despite its structural complexity. This size-enabled transparency extends beyond the visible range.

The crystallized samples also demonstrate excellent infrared transmittance (~80% across 800–4400 nm), with minimal variation between different crystallization temperatures. This stability originates from nanocrystalline grain sizes being substantially smaller than incident wavelengths (*λ* > 800 nm), satisfying the Rayleigh scattering criterion that suppresses grain-boundary scattering. Sub-wavelength grain size variations negligibly influence optical performance in the NIR-MIR spectral range. The small absorption peaks at 2500–3000 nm and 4250 nm correspond to hydroxyl (−OH) and CO_2_ vibration modes, respectively. The infrared cut-off wavelength (5% transmittance threshold) extends to ~7.3 μm for crystallized samples, exceeding that of glass counterparts (~6.7 μm). In addition to its broadband transparency, the material exhibits tunable refractive index upon crystallization.

LYTZA glass exhibits a high refractive index (*n*_d_) of 1.91 at 587.6 nm, which increases with crystallization temperature. Figure 3d shows the refractive index (*n*) dispersion of the crystallized samples. In oxide glass systems, the refractive index is governed by three critical factors: material density, oxygen packing density (OPD), and polarizability of oxygen ions (${\alpha }_{{{\mbox{O}}}^{2-}}$)^30,41,42^. As shown in Fig. 1b, the crystallized samples show a substantial density increase due to volume contraction, which typically reduces light propagation speed, thereby elevating the refractive index. Furthermore, Supplementary Fig. 4b reveals an inverse correlation between molar volume (*V*_m_) and OPD with rising crystallization temperature. The decreased *V*_m_ values indicate densified glass networks, while enhanced OPD corresponds to slower light transmission, and both align with the observed increase in refractive index. High ${\alpha }_{{{\mbox{O}}}^{2-}}$ can enhance the dielectric polarization of glass, leading to an increase in dielectric constant and refractive index. As calculated via the Lorentz–Lorenz equation (${\alpha }_{{{\mbox{O}}}^{2-}}$ values in Supplementary Fig. 4c), within the 900–1050 °C crystallization temperature range, the *V*_m_ reduction becomes less pronounced while the refractive index demonstrates a positive correlation with ${\alpha }_{{{\mbox{O}}}^{2-}}$. These observations are consistent with previous reports on oxide systems^30,42,43^.

High-index materials typically exhibit low Abbe numbers (high dispersion), degrading imaging clarity and color fidelity in optics like AR/VR devices, microscopes, and lenses. This necessitates additional low-dispersion lenses for chromatic correction, complicating optical system design. Hence, materials combining high refractive index with low dispersion are highly sought after. The LYTZA glass crystallized at 900–1000 °C shows a slight increase in refractive index accompanied by a remarkable enhancement in *ν*_d_ (Fig. 3d). Comparative analysis in Fig. 3e demonstrates that commercial optical glasses strictly follow the inverse *n*_d_–*ν*_d_ correlation; those with *n*_d_ > 1.9 universally show *ν*_d_ confined to 20–40. Remarkably, the sample crystallized at 900 °C achieves *n*_d_ = 1.93 and *ν*_d_ = 60.94. To the best of our knowledge, such a combination (*n*_d_ > 1.9, *ν*_d_ > 60) has rarely been reported in optical materials and is highly desired in realizing the achromatic optical lenses of thin size. Confocal testing (Fig. 3f) further confirms its low optical dispersion, with a focal shift of only 6% across the 470–650 nm wavelength range. This result inverts the conventional inverse relationship and outperforms established high-index systems, including 30.8La_2_O_3_-69.2TiO_2_ glass (15)^44^, La_2_O_3_-Nb_2_O_5_-Ta_2_O_5_ glass (26–33)^45^, 30.8La_2_O_3_-64.2TiO_2_-5Bi_2_O_3_ glass (26.9)^46^, and transparent Gd_2_Sn_2_O_7_ pyrochlore ceramics (32.7)^47^. The enhanced *ν*_d_ is observed in samples crystallized at 900 °C, which contain three distinct nanoscale crystalline phases (~10 nm grains, estimated by the Scherrer equation, Fig. 1e) together with a residual glass matrix. Raising the crystallization temperature correlates with grain coarsening and a return of *ν*_d_ to conventional levels. Notably, the sample crystallized at 900 °C still maintains 80.8% transmittance at 700 nm, positioning it as a promising candidate for precision optical systems, including camera lenses, laser devices, and AR/VR components.

**Fig. 4:**
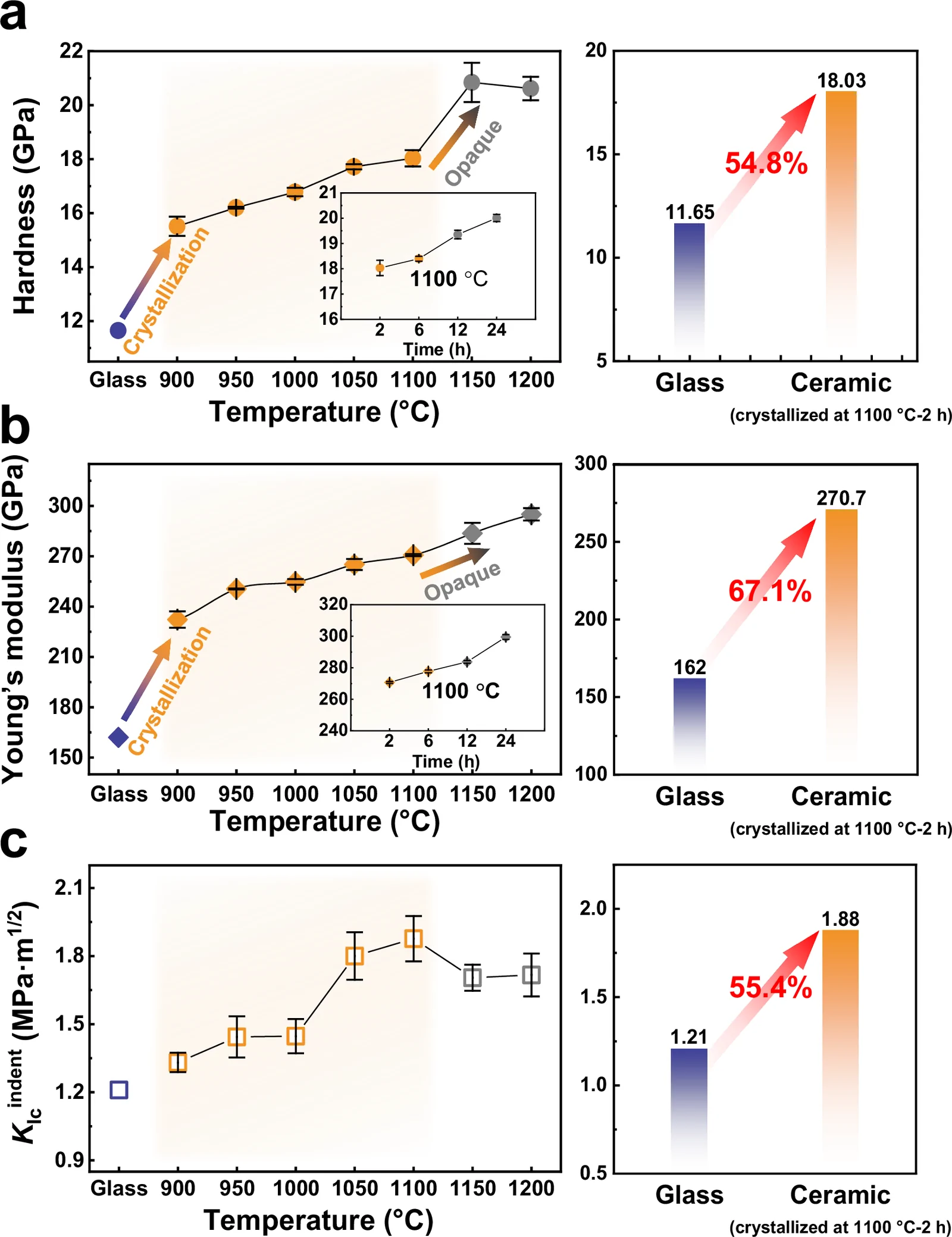
Mechanical properties of multiphase nanocrystalline ceramics. **a**, **b** Hardness and Young’s modulus of samples crystallized from LYTZA glass at 900–1200 °C for 2 h (The insets show samples crystallized from LYTZA glass at 1100 °C for 2–24 h). Data are presented as mean values, and error bars represent standard deviation from *n* = 5 independent indentations (technical replicates). **c** Indentation fracture toughness *K*_Ic_^indent^ of samples crystallized from LYTZA glass at 900–1200 °C for 2 h. Data are presented as mean values, and error bars represent standard deviation from *n* = 10 independent indentations (technical replicates). The light yellow background in (**a**–**c**) highlights the temperature range 900–1100 °C, where the crystallized samples remain transparent. Source data are provided as a Source Data file.

In addition to its optical performance, the nanocrystalline structure is associated with mechanical properties. Figure 4a, b demonstrate enhanced hardness (from 11.65 to 15.51–20.84 GPa) and Young’s modulus (from 162 to 232.3–299.5 GPa) in crystallized LYTZA glass under elevated temperatures (900–1200 °C) for 2 h and extended durations (2–24 h) at 1100 °C. The synergistic enhancement of mechanical properties is consistent with inverse Hall–Petch behavior, which dominates at grain sizes below 30 nm with hardness increasing as grains coarsen^48–51^. For samples crystallized below 1150 °C, grain sizes remain within the 10–30 nm range (Fig. 1e), and their progressive coarsening with increasing temperature enables controlled hardness enhancement within this regime. At 1200 °C, the grain size reaches 60.4 nm (Fig. 1e), leading to a slight hardness decline from 20.84 to 20.62 GPa, aligning with classical Hall–Petch behavior^52–54^. This dual-regime mechanical response illustrates grain size-dependent strengthening mechanisms in nanocrystalline ceramics.

As shown in Fig. 4a, b, crystallizing the LYTZA glass at 1100 °C for 2 h results in a 54.8% increase in hardness (18.03 GPa) and a 67.1% enhancement in Young’s modulus (270.7 GPa), demonstrating substantially improved mechanical properties relative to the glass precursor. Comparative analysis with reported transparent ceramics (Supplementary Table 4) shows that the achieved hardness ranks among the higher values, surpassing those of MgAl_2_O_4_^55^ (~16 GPa), ZnGa_2_O_4_^56^ (10.56 ± 0.08 GPa), and Y_2_O_3_^57^ (8.8 ± 0.2 GPa). The Young’s modulus (270.7 GPa) is comparable to that of MgAlON^58^ (288 GPa) and exceeds most reported values for transparent ceramics (Supplementary Table 4).

Fracture toughness (*K*_Ic_), representing a material’s resistance to crack propagation, was evaluated using the Vickers indentation fracture (IF) method. Due to sample size limitations that precluded the use of the Single Edge Precracked Beam (SEPB) method, the values are explicitly denoted as *K*_Ic_^indent^ to indicate that they were obtained via the IF method. As shown in Fig. 4c, crystallization at 1100 °C for 2 h increases *K*_Ic_^indent^ by 55.4% (from 1.21 to 1.88 MPa·m^1/2^). This value substantially exceeds those of most oxide glasses reported to date (typically below 1 MPa·m^1/2^)^27^ and approaches that of the LYTZA glass paracrystallized under high‑pressure (15 GPa, 1000 °C) toughening (1.95 MPa·m^1/2^)^59^. It is also comparable to that of ZnGa_2_O_4_^56^ transparent ceramics (1.83 MPa·m^1/2^) and falls within the range of fracture toughness values reported for most transparent ceramics (0.65–4.45 MPa·m^1/2^, Supplementary Table 4). The enhanced fracture toughness is partly attributed to densification with increasing crystallization temperature. Additionally, the grain boundaries of the three distinct nanoscale crystal phases hinder crack propagation, further improving fracture toughness. However, when the crystallization temperature is increased to 1150 °C, *K*_Ic_^indent^ decreases to 1.71 MPa·m^1/2^. This decline is associated with excessive grain growth and the development of heterogeneous residual stresses arising from mismatched thermal expansion coefficients among the different phases^60^. When these thermally induced stresses surpass a critical threshold under external loading, they can facilitate crack initiation through localized stress concentration^61,62^, potentially reducing fracture toughness.

These results demonstrate that the multiphase nanoceramic fabricated via multiphase interface locking, with its nanocrystalline grain size (<20 nm), achieves a favorable balance of mechanical properties, including hardness, modulus, and fracture toughness, while maintaining high optical transparency. The grain size-dependent strengthening mechanisms elucidated here further establish the potential of such materials for applications requiring combined optical and mechanical performance.

## Discussion

Multiphase interface locking during the crystallization of LYTZA glass precursors enables the synthesis of transparent nanoceramics composed of La(Al_1/3_Ti_2/3_)O_3_, Y_2_(Zr_0.6_Ti_0.4_)_2_O_7_, and Al_2_O_3_. The fully dense nanoceramic, comprising two cubic phases and an Al_2_O_3_ intergranular phase, exhibits a remarkably fine grain size down to <20 nm with a uniform size distribution. Owing to its nanocrystalline microstructure, the material achieves favorable optical performance with high transmittance of nearly 70% in the UV–Vis range, along with enhanced mechanical properties (hardness: 18.03 GPa, Young’s modulus: 270.7 GPa, fracture toughness: 1.88 MPa·m^1/2^). Notably, samples crystallized at 900 °C for 2 h exhibit a high refractive index (1.93) and an exceptionally high Abbe number (60.94), demonstrating superior optical characteristics. The tailorable crystallization behavior of MPEGs at different temperatures not only enables the fabrication of polyphase transparent nanoceramics with nanocrystalline grains but also opens new avenues for developing multifunctional transparent ceramics via the glass crystallization route.

## Methods

### Materials synthesis

La_2_O_3_ (99.99%, Beijing Hulk Technology Co., Ltd., China), Y_2_O_3_ (99.99%, Aladdin Reagent (Shanghai) Co., Ltd., China), TiO_2_ (99.99%, Sinopharm Chemical Reagent Co., Ltd), ZrO_2_ (99.99%, Aladdin Reagent (Shanghai) Co., Ltd., China), and Al_2_O_3_ (99.99%, Aladdin Reagent (Shanghai) Co., Ltd., China) powders were weighed according to the molar ratio of 18.77La_2_O_3_-4.83Y_2_O_3_-28.22TiO_2_-8.75ZrO_2_-39.43Al_2_O_3_. The powders were mixed with absolute ethanol and subjected to ball milling for two cycles to ensure homogeneity. The resulting paste was dried in a vacuum drying oven at 75 °C for 45 min. The dried powder was then uniaxially pressed at a pressure of 10 MPa for 2 min to form discs with a diameter of 20 mm, which were subsequently broken into smaller fragments. Approximately 50 mg of these fragments were introduced into an aerodynamic levitation furnace, where an oxygen flow was adjusted to levitate the sample. The sample was then melted using a triple-path CO_2_ laser heating system. Once the sample was completely molten and stable in suspension for several tens of seconds, the laser was turned off, allowing the sample to rapidly cool and solidify at room temperature into glass spheres with diameters ranging from 3 to 5 mm.

The glass spheres were lapped and polished into discs to eliminate surface defects such as bubbles. Subsequently, these discs were subjected to a single heat treatment at a heating rate of 3 °C min^−1^, within the temperature range of 900–1200 °C for 2 h, followed by an extended crystallization at 1100 °C for 6, 12, and 24 h to obtain crystallized samples. A schematic of the sample preparation process is shown in Supplementary Fig. 7.

#### Characterizations

The thermal behavior of the LYTZA glass was analyzed using a Simultaneous Thermal Analyzer (DSC–TGA, SDT 650, Waters, America) from room temperature to 1200 °C with argon as the purging gas at a heating rate of 10 °C min^−^^1^. The phase composition and grain size of the samples were characterized using an X-ray powder diffractometer (XRD, Smartlab 9, Rigaku Corporation, Tokyo, Japan) with Cu Kα radiation. The instrument was operated at a testing voltage of 45 kV and a current of 40 mA. The scanning range for the 2*θ* angle was from 5 to 90°, with a scanning speed of 3° min^−1^ and a step size of 0.02°. The proportion of crystalline phases and the unit cell parameters of the samples were obtained by combining the XRD results and applying the Rietveld method for full spectrum fitting (the least squares method was used for fitting). The grain size was estimated using the Scherrer formula^33^ when stress effects were not considered:

1 $$D=\frac{{\mbox{K}}\gamma }{\beta \cos \theta }$$

where *D* is the grain size of the material, *β* is the FWHM of the diffraction peak of the tested sample, *K* is the Scherrer constant (0.89), *θ* is the diffraction angle, and *γ* is the X-ray wavelength (0.154059 nm). High-angle annular dark-field (HAADF)–STEM images and EDS mappings were observed using a Spherical Aberration Correction Transmission Electron Microscope (TEM, JEM ARM200 F, JEOL, Japan) operated at 200 kV. The density of the samples was measured using an electronic balance (ME204, Mettler-Toledo, Switzerland) based on the Archimedes principle. Defect-free samples were selected, and the density was determined by averaging ten measurements for each sample. The theoretical density of the multiphase ceramic was calculated based on the rule of mixtures using the nominal phase composition. The theoretical densities of the individual phases were obtained from the corresponding PDF cards: 6.325 g cm^−3^ for LaAlO_3_, 6.472 g cm^−3^ for LaTiO_3_, 5.333 g cm^−3^ for Y_2_(Zr_0.6_Ti_0.4_)_2_O_7_, and 3.665 g cm^−3^ for Al_2_O_3_. The density of the La(Al_1/3_Ti_2/3_)O_3_ phase was estimated as the weighted average of LaAlO_3_ and LaTiO_3_ according to the B‑site occupancy (1/3 Al and 2/3 Ti), yielding a value of 6.423 g cm^−3^. Using the nominal volume fraction of each phase (La(Al_1/3_Ti_2/3_)O_3_: Y_2_(Zr_0.6_Ti_0.4_)_2_O_7_: Al_2_O_3_ = 49.7%: 6.39%: 43.91%), the overall theoretical density was calculated to be 5.142 g cm^−3^.

The in-line transmittance (T) of the samples (each 1.0 mm thick) was measured across the ultraviolet–visible–near-infrared (UV–Vis–NIR) region (200–2500 nm) using a spectrophotometer (Cary 5000, Agilent Technologies, America) in its standard transmission configuration. A dual-beam optical arrangement with 1 mm apertures on both the reference and sample beams was employed, and the sample was precisely secured in the compartment with air as the baseline. In this calibrated setup, the sample was positioned at the standard height (Z-axis ≈ 20 mm) within the sample chamber, where the factory-calibrated light source and detector are fixed relative to each other. This geometry ensures that only directly transmitted light within a small solid angle is collected, minimizing scattered light contributions. For the mid-infrared (MIR) region (2500–8000 nm), in-line transmittance was measured using a Fourier transform infrared spectrometer (FTIR Excalibur 3100, Varian, America). Reflectance (R) of the sample was tested with a self-built micro-area spectroscopy system (300–800 nm), using air and an aluminum mirror as the references for transmittance and reflectance, respectively. Absorptance (A) is given by the formula A = 1 − T − R. The refractive indices of the samples were characterized using an ellipsometer (SENTECH SE850 DUV, Germany) in the 300–900 nm spectral range. The Abbe number (*ν*_d_) was calculated for each sample using the dispersion formula:

2 $${v}_{{\mbox{d}}}=\frac{{n}_{{\mbox{d}}}-1}{{n}_{{\mbox{F}}}-{n}_{{\mbox{C}}}}$$

where *n*_d_, *n*_F_, and *n*_C_ represent the refractive indices measured at the Fraunhofer D-line (587.6 nm, Na), F-line (486.1 nm, Hβ), and C-line (656.3 nm, Hα), respectively. The oxygen packing density (OPD) of the glass was calculated using the partial molar volume of oxygen (*V*_O_) and the radius of an oxygen ion *R*_O_. The partial molar volume of oxygen (*V*_O_) was derived by subtracting the cationic contributions from the molar volume (*V*_m_), where *V*_m_ represents the molar weight of the glass divided by its density. Based on Shannon’s ionic radius data (1.4 Å for O^2−^), the theoretical molar volume of oxygen ions ${V}_{{{\mbox{O}}}^{2-}}$ was adopted as 6.92 cm^3 ^mol^−1^. The calculations for *V*_m_, *V*_O_ and OPD were performed using the following formulas:

3 $${V}_{{\mbox{m}}}=\frac{\sum {x}_{i}{M}_{i}}{\rho }$$

4 $${{{\rm{OPD}}}}=\frac{{V}_{{{\mbox{O}}}^{2-}}}{{V}_{{\mbox{O}}}}=\frac{\frac{4}{3}{{{\rm{\pi }}}}{N}_{A}{R}_{{\mbox{O}}}^{3}}{\frac{\sum {x}_{i}{M}_{i}}{\rho \sum {x}_{i}{n}_{i}}}$$

where *x*_*i*_ and *M*_*i*_ denote the molar percentage and molar mass of each oxide component respectively, *ρ* is the glass density, *n*_*i*_ is the number of oxygen ions in each oxide.

The electronic polarizability of oxygen ions (${\alpha }_{{{\mbox{O}}}^{2-}}$) was calculated via the Lorentz–Lorenz equation incorporating refractive index and density data:

5 $$\left(\frac{{n}^{2}-1}{{n}^{2}+2}\right)\cdot \left(\frac{M}{\rho }\right)=\frac{4{{{\rm{\pi }}}}\left(\sum {\alpha }_{{\mbox{i}}}+{N}_{{{\mbox{O}}}^{2-}}\cdot {\alpha }_{{{\mbox{O}}}^{2-}}\right){N}_{A}}{3}$$

where *M* is the molecular weight of the glass composition, *N*_*A*_ is the Avogadro’s number, ${N}_{{{\mbox{O}}}^{2-}}$ represents the total number of oxygen ions, and ${\alpha }_{{\mbox{i}}}$ corresponds to the cation polarizabilities (La^3+^: 1.04 Å^3^, Y^3+^: 0.544 Å^3^, Ti^4+^: 0.184 Å^3^, Zr^4+^: 0.357 Å^3^ and Al^3+^: 0.054 Å^3^)^63^. The hardness and Young’s modulus of the samples were measured using a nanoindenter (G200, Keysight Technologies, USA) with a penetration depth of 2000 nm. The hardness and Young’s modulus of the samples were derived based on the Oliver–Pharr model. The hardness (*H*_V_) was calculated using the following formula:

6 $${H}_{{\mbox{V}}}=\frac{P}{A}$$

where *P* is the indentation load, and *A* is the projected contact area under the indenter. Young’s modulus (*E*) was obtained from the relation:

7 $$E=\frac{\sqrt{{{{\rm{\pi }}}}}}{2\beta }\frac{S}{\sqrt{A}}$$

where *S* is the elastic contact stiffness and *β* is a correction factor (approximately 1.034 for a Berkovich indenter). The contact stiffness *S* was determined as:

8 $$S=\lambda m{({h}_{\max }-{h}_{f})}^{m-1}$$

where λ and m are fitting parameters, *h*_*max*_ is the maximum indentation depth, and *h*_*f*_ is the residual depth after unloading. At least five indentations were performed on each sample using a diamond (Berkovich) indenter, and the average value was reported.

The fracture toughness was evaluated using a Vickers hardness tester (HMAS-D1000Z, Shanghai, China) under a load of 4.9 N with a dwell time of 10 s. During the test, a well‑defined indentation and associated cracks are produced on the sample surface. Supplementary Fig. 6 confirms that 4.9 N is sufficient to produce well‑defined cracks without ring cracks, while 9.8 N causes ring crack formation. Thus, 4.9 N was chosen as the optimal load. By measuring the crack lengths and combining them with the corresponding load, hardness, and Young’s modulus, the indentation fracture toughness (*K*_Ic_^indent^) was calculated using the following empirical relation^64^:

9 $${K}_{{{{\rm{Ic}}}}}^{{{{\rm{indent}}}}}=0.016{\left(\frac{E}{{H}_{{\mbox{V}}}}\right)}^{0.5}\frac{P}{{c}^{1.5}}=0.23{\left({EP}\right)}^{0.5}\frac{a}{{c}^{1.5}}$$

where *E* is the Young’s modulus, *P* is the applied load, *c* is the average crack length from the indentation center to the crack tip, and *a* is the half‑diagonal length of the Vickers impression. To enhance measurement accuracy, at least ten indentations were made in different areas of each sample using a diamond indenter. All measurements were conducted at 25 °C and 50% relative humidity.

### Reporting summary

Further information on research design is available in the Nature Portfolio Reporting Summary linked to this article.

## Supplementary information

Supplementary Information

Reporting Summary

Transparent Peer Review file

## Data Availability

All data generated in this study have been deposited in the Figshare database under accession code https://doi.org/10.6084/m9.figshare.32073057. Source data are provided with this paper.
