# Supplementary material for: Breaking the grain size limit of glass-crystallized transparent nanoceramics via multi-phase interface locking
Source: Nat Commun. 2026 Aug 15;17:9825. doi: 10.1038/s41467-026-76601-5 (PMC13575170; doi:10.1038/s41467-026-76601-5)
Supplement: Supplementary file 1 [file 41467_2026_76601_MOESM1_ESM.pdf]

SUPPLEMENTARY INFORMATION

Breaking the grain size limit of glass-crystallized transparent nanoceramics via multi-phase interface locking

Wenlong Xu[1], Yongchang Guo[2], Haohan Fan[3], Yuanfeng Liu[4], Zhibiao Ma[1], Linghan Bai[1], Ruyu Fan[1], Tiannan Zhang[1], Guoguo Zhang[1], Yuhang Chen[1], Shijiao Zhang[1], Chuanbao Liu[1,*], Binghui Ge[3,*], Jingbo Sun[4,*], Yanhao Dong[4,*], and Jianqiang Li[1,5,*]

[1]School of Materials Science and Engineering, University of Science and Technology Beijing, Beijing 100083, China

[2]University of Chinese Academy of Sciences, Beijing 100049, China

[3]State Key Laboratory of Opto-Electronic Information Acquisition and Protection Technology, Leibniz International Joint Research Center of Materials Sciences of Anhui Province, Institutes of Physical Science and Information Technology, Anhui University, Hefei 230601, China

[4]State Key Lab of New Ceramic Materials, School of Materials Science and Engineering, Tsinghua University, Beijing 100084, China

[5]Beijing Key Laboratory for Advanced Powder Metallurgy and Particulate Materials, University of Science and Technology Beijing, Beijing 100083, China

[*]Correspondance: jianqiangli@ustb.edu.cn (J.L.), dongyanhao@tsinghua.edu.cn (Y.D.), jingbosun@tsinghua.edu.cn (J.S.), bhge@ahu.edu.cn (B.G.), cbliu@ustb.edu.cn (C.B.)

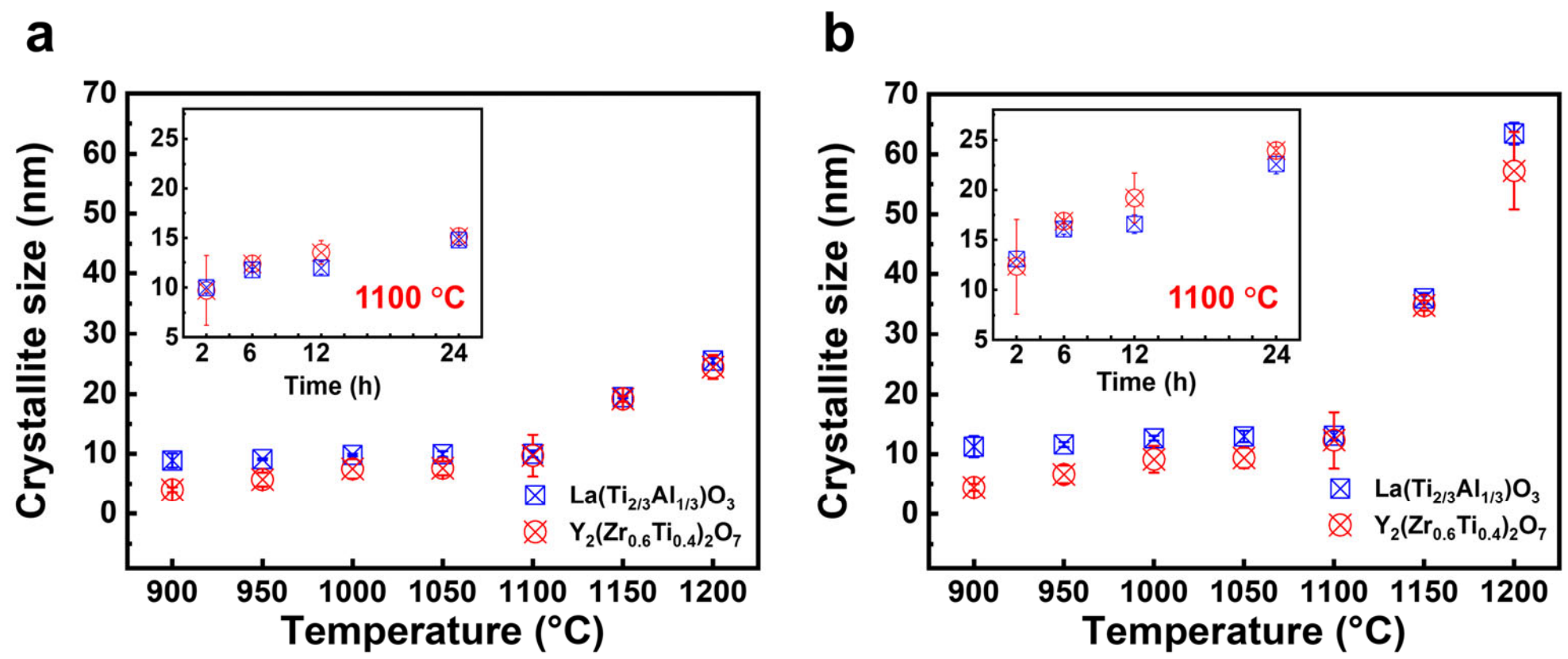

Supplementary Fig. 1 Comparison of fitted grain sizes after correcting for instrumental broadening. a, Fitted grain size without removal of instrumental broadening effects. **b,** Fitted grain size after removal of broadening effects. Source data are provided as a Source Data file.

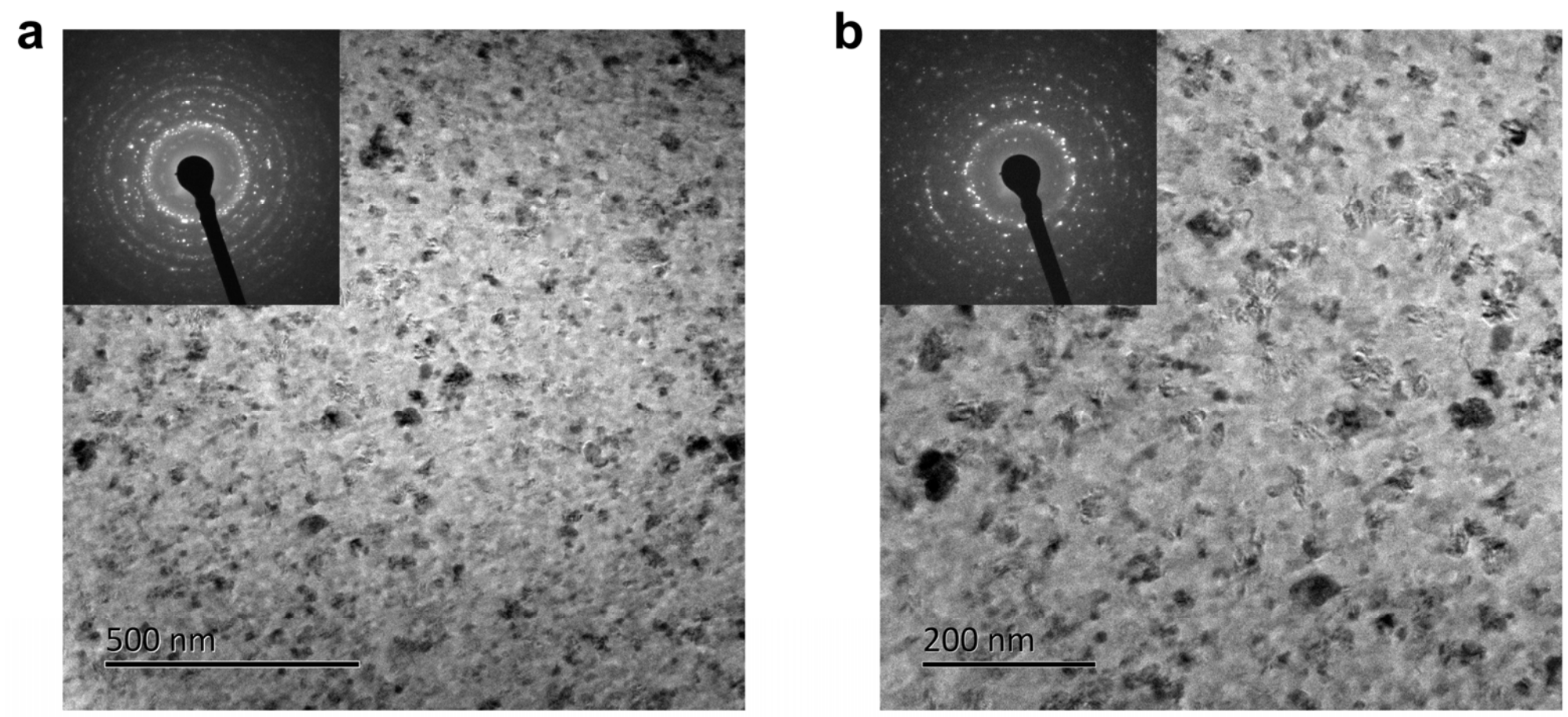

Supplementary Fig. 2 Microstructure and crystallinity of the fully crystallized material. a, Bright-field TEM image showing the overall morphology (scale bar: 500 nm). **b,** Higher-magnification TEM image revealing finer structural details (scale bar: 200 nm). The selected area electron diffraction (SAED) pattern (inset) exhibits sharp reflections from three distinct phases with no diffuse halos characteristic of an amorphous phase, confirming complete crystallization.

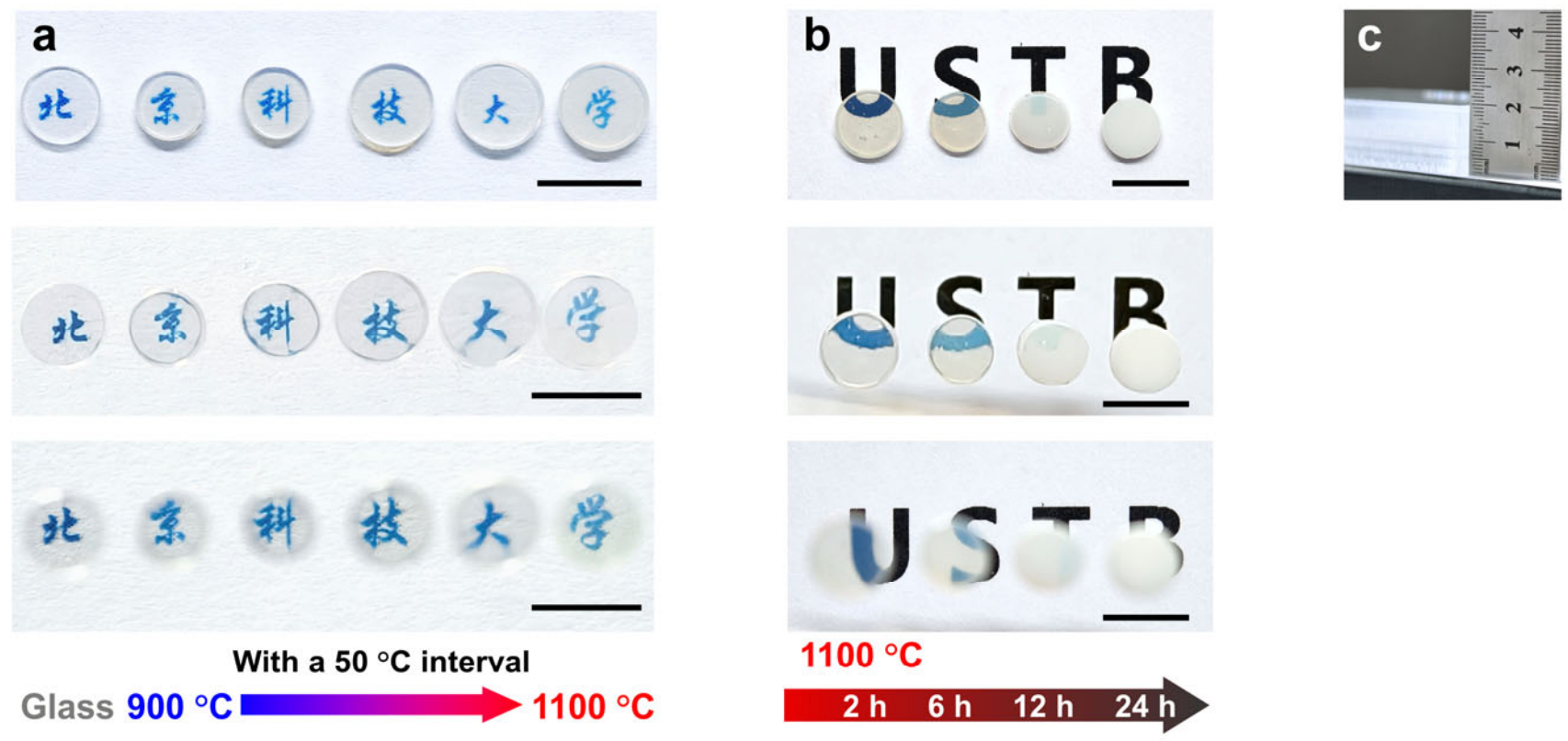

Supplementary Fig. 3 Sample photographs and imaging setup. a, LYTZA glass samples crystallized at 900–1100 °C for 2 h. For each sample, three photographs are shown from top to bottom: placed directly on the background, placed on a polycarbonate board 2 cm above the background using a standard lens, and using a telephoto lens. **b,** LYTZA glass samples crystallized at 1100 °C for 2–24 h, arranged in the same order as in **(a)**. **c,** Photograph of the polycarbonate board used for sample elevation. Scale bars in **(a)** and **(b)** are 5 mm.

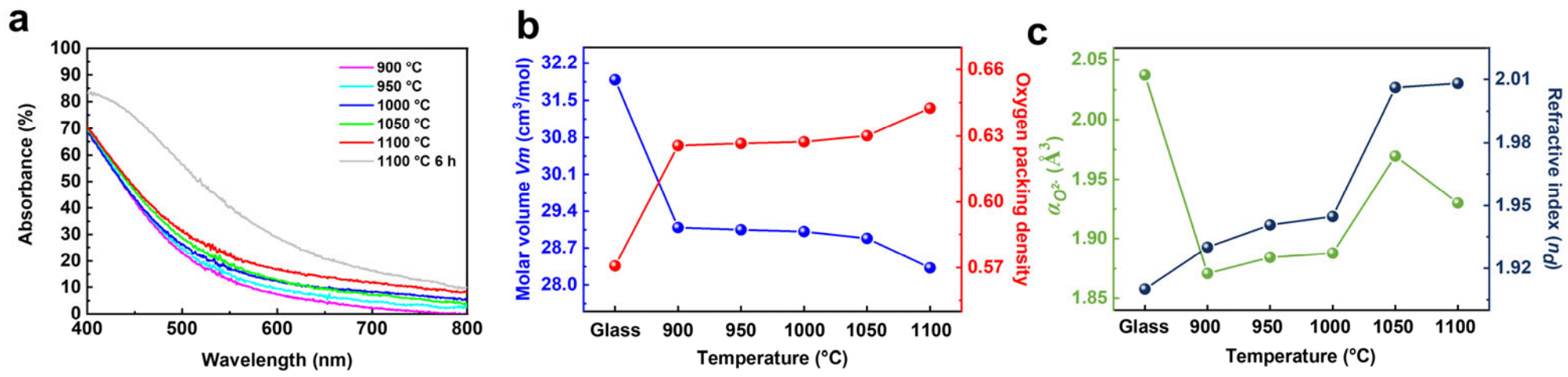

Supplementary Fig. 4 Optical absorption and refractive index-related properties of crystallized LYTZA glass. a, Absorbance spectra in the 400–800 nm range of LYTZA glass crystallized at 900–1100 °C for 2 h and at 1100 °C for 6 h. **b,** Changes in molar volume (V_{m}) and oxygen packing density (OPD) with crystallization temperature. **c,** Changes in oxygen ion polarizability ($\alpha_{\mathrm{O^{2-}}}$) and refractive index (n_{d}) with crystallization temperature. Source data are provided as a Source Data file.

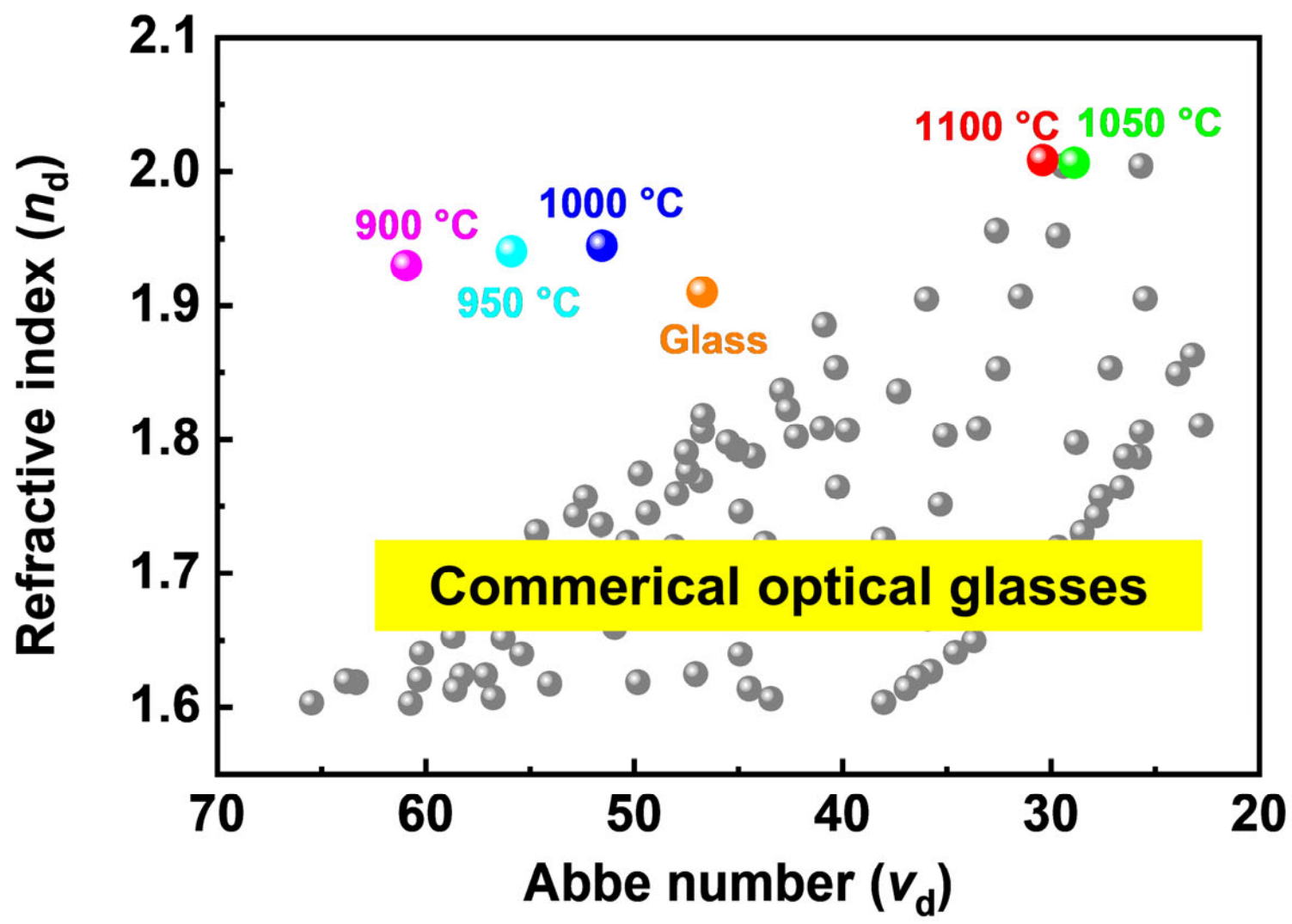

Supplementary Fig. 5 Refractive index at 587.6 nm versus Abbe number (v_d) for the prepared samples compared with commercial optical glasses (this figure is an enlarged version of Fig. 3e). All grey data points representing commercial optical glasses were reproduced from Fig. 4 of Yoshimoto et al.[S1]. Source data are provided as a Source Data file.

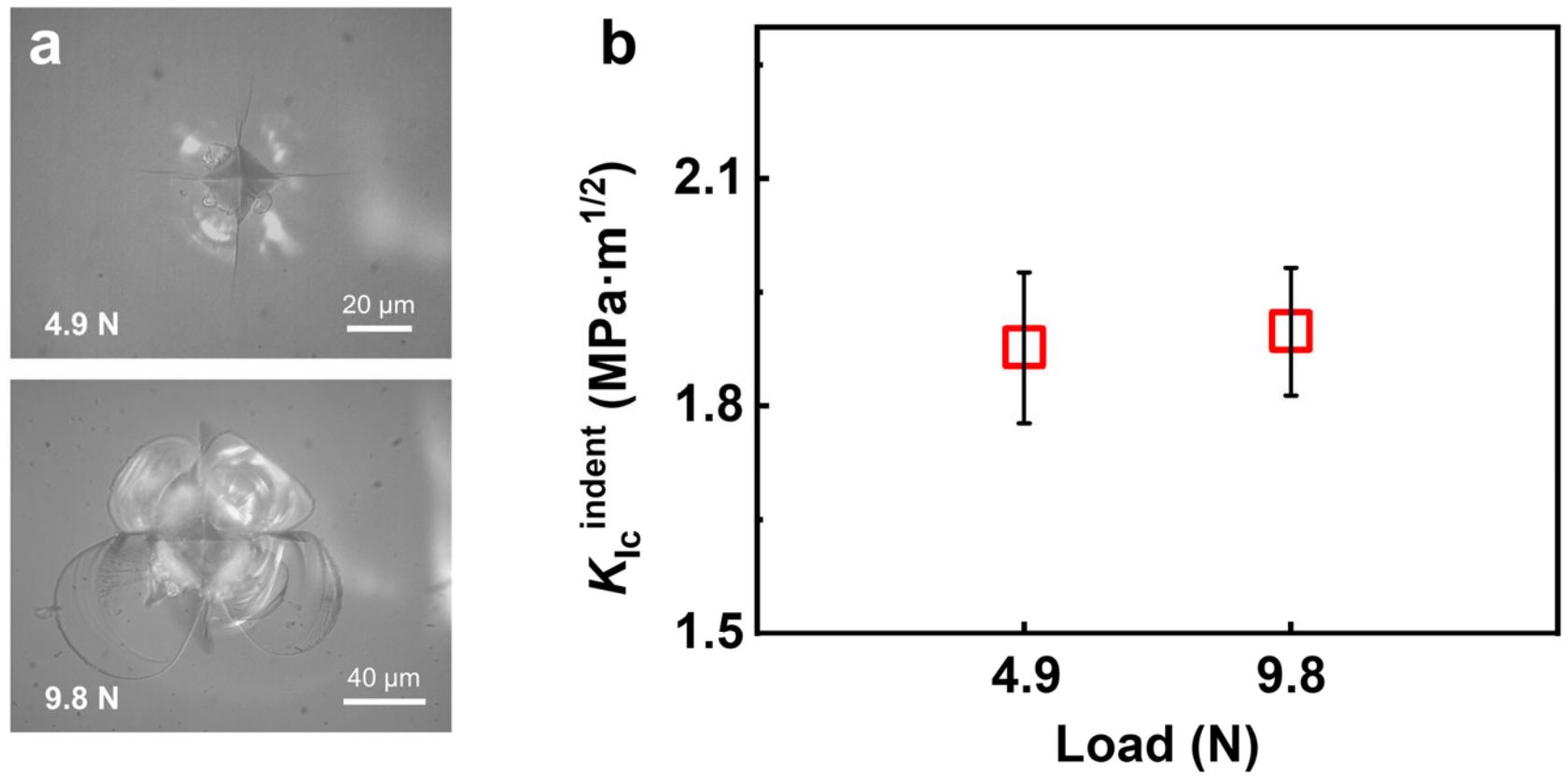

Supplementary Fig. 6 Load dependence of indentation fracture behavior. a, Representative optical micrographs of indentations on the sample crystallized at 1100 °C for 2 h, performed at 4.9 N and 9.8 N. At the higher load of 9.8 N, the sample became prone to surface ring crack formation. **b,** Indentation fracture toughness (K_{Ic}^{indent}) of the sample crystallized at 1100 °C for 2 h, measured under loads of 4.9 N and 9.8 N. Source data are provided as a Source Data file.

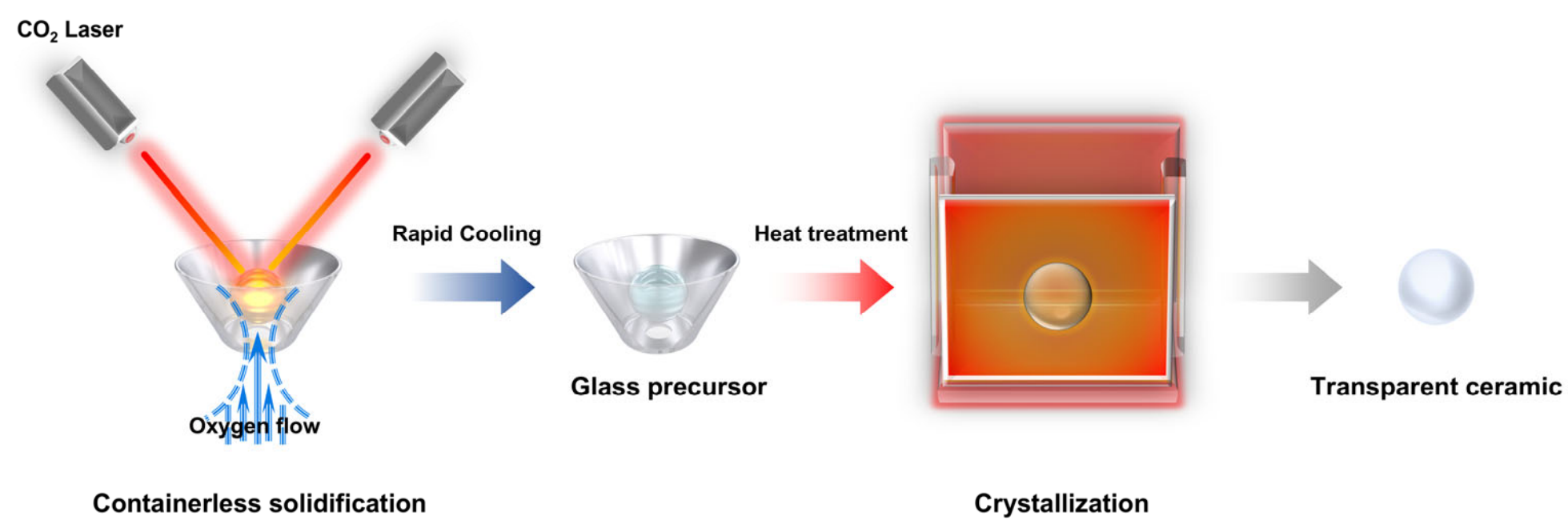

Supplementary Fig. 7 Schematic diagram of sample preparation via glass crystallization route.

Supplementary Table 1 Comparison of grain size fitting results for samples crystallized above 1100 °C.

Crystallization temperature	Crystalline phase	Fitted grain size (uncorrected for instrumental broadening)	Fitted grain size (corrected for instrumental broadening)
1100 °C G_{avg}	$La(Al_{1/3}Ti_{2/3})O_3$	9.99 nm	13.06 nm
	$Y_2(Zr_{0.6}Ti_{0.4})_2O_7$	9.71 nm	12.32 nm
1150 °C G_{avg}	$La(Al_{1/3}Ti_{2/3})O_3$	19.52 nm	35.93 nm
	$Y_2(Zr_{0.6}Ti_{0.4})_2O_7$	19.21 nm	34.77 nm
1200 °C G_{avg}	$La(Al_{1/3}Ti_{2/3})O_3$	25.50 nm	63.47 nm
	$Y_2(Zr_{0.6}Ti_{0.4})_2O_7$	24.48 nm	57.25 nm

Supplementary Equation (1) Instrument half-height and width correction curve formula:

$$\beta_{instr} = 0.09531509 + 3.237096 \times 10^{-3} \times (2\theta) - 1.750616 \times 10^{-5} \times (2\theta)^2 \quad (1)$$

Supplementary Table 2 Composition (from STEM-EDS) of the region in Fig. 2d.

Element	La	Y	Ti	Zr	Al	O
Content (atomic percent)	9.46	1.83	6.05	1.47	16.27	64.92

Supplementary Table 3 Comparison of transparent ceramics derived from glass crystallization: material systems, processing routes, achieved grain sizes, and optical transmittance.

Material system	Processing route	Grain size	Optical property	
			Transmittance	Refractive index
$Bi_{0.8}Nb_{0.8}Te_{2.4}O_8$ [S2]	510 °C, 1.5 h	8 μm	74% (3–5 μm)	2.24 ± 0.05 (1064 nm)
$Sr_{1+x/2}Al_{2+x}Si_{2-x}O_8$ ($0<x\leqslant 0.4$) [S3]	1020 °C, 3 h	5–20 μm	T_{max}~90% (Vis–NIR)	1.59
α-$BaAl_4O_7$/β-$BaAl_4O_7$ [S4]	1000 °C, 2 h/ 1100 °C, 24 h	0.5–2 μm/ 1–5 μm	59.2%/39.5% (633 nm)	1.649/1.623 (514.5 nm)
β-$BaAl_4O_7$−$BaAl_2O_4$ [S5]	1100 °C, 20 h	1.4–3.2 μm	40–60% (1200 nm)	-
$Sr_3Al_2O_6$ [S6]	840 °C, 5 h	0.5–2 μm	T_{max}~87.4±0.5% (300 nm–6 μm)	1.70 ± 0.02
$Y_2CaAl_4SiO_{12}$ [S7]	1050 °C, 10 h/ 1150 °C, 10 h/ 1250 °C, 10 h	21.4 ± 6.9 nm/ 150.8 ± 51.3 nm/ 760 ± 250 nm	28% (450 nm, 1250 °C)	-
$Ca_3Al_2Si_3O_{12}$ [S8]	>10 GPa, 1400 °C, 2 h	30–50 nm	T_{max}~86.7% (400–800 nm)	-
$LaAlO_3$/t-ZrO_2 [S9]	1200 °C, 2 h	40 nm (95.2% crystallinity)	55% (800 nm)	-
$Lu_3Al_5O_{12}$−Al_2O_3 [S10]	1100 °C, 2 h	65.7 ± 2.3 nm	82% (780 nm)	-
$GdAlO_3$−Al_2O_3 [S11]	1000 °C, 2 h	42 ± 2 nm	77% (780 nm)	-
$Y_3Al_5O_{12}$−Al_2O_3 [S12]	1100 °C, 2 h	30 nm	T_{max}~87% (NIR)	1.77 (589 nm)
This work: $La(Al_{1/3}Ti_{2/3})O_3$−$Y_2(Zr_{0.6}Ti_{0.4})_2O_7$−$Al_2O_3$	1100 °C, 2 h	16 nm	69.5% (700 nm)	2.01 (587.6 nm)

Supplementary Table 4 Mechanical properties of transparent ceramics with different material systems: hardness, Young's modulus, and indentation fracture toughness.

**Material system**	**Hardness (GPa)**	**Young's modulus (GPa)**	**Indentation fracture toughness (MPa·m$^{1/2}$)**
$MgAl_2O_4$[S13]	16	210 ± 10	1.8 ± 0.1
$Mg_{0.27}Al_{2.58}O_{3.73}N_{0.27}$ (MgAlON)[S14]	15.1	288	-
$Y_3Al_5O_{12}$[S15]	12.5	221	2.21
$Y_3Al_5O_{12}$[S16]	22	271	2.23
$Y_3Al_5O_{12}$[S16]	15	212	4.45
AlON[S17]	15.6-16.6	-	1.6-2.0
0.1 at% Cr:Y_2O_3[S18]	10.10 ± 0.04	-	1.11 ± 0.05
$ZnGa_2O_4$[S19]	10.56 ± 0.08	227.04	1.83 ± 0.08
CaF_2:Yb[S20]	3.2 ± 0.07	-	0.65 ± 0.12
8YSZ[S21]	12.9±0.1	-	1.30±0.02
$Ca_3Al_2Si_3O_{12}$[S22] (paracrystallization)	7.7 ± 0.09	124.5 ± 1.4	1.99±0.06
$18.77La_2O_3-4.83Y_2O_3-28.22TiO_2-8.75ZrO_2-39.43Al_2O_3$[S22] (paracrystallization)	9.5±0.2	168.5±1.9	1.95±0.08
$LaAlO_3/tZrO_2$[S9] (95.2% crystallinity)	19.05	-	2.64
$Sr_3Al_2O_6$[S6]	10.5	150	-
$Y_3Al_5O_{12}-Al_2O_3$[S19]	23.6	281	-
$GdAlO_3-Al_2O_3$[S11]	22	284.7	-
This work: $La(Al_{1/3}Ti_{2/3})O_3-Y_2(Zr_{0.6}Ti_{0.4})_2O_7-Al_2O_3$	18.4	270.7	1.88

Supplementary References

S1. Yoshimoto, K. Low phonon energies and wideband optical windows of La_2O_3-Ga_2O_3 glasses prepared using an aerodynamic levitation technique. Sci. Rep. **7**, 45600 (2017).

S2. Bertrand, A. *et al.* Scalable and Formable Tellurite-Based Transparent Ceramics for Near Infrared Applications. *Adv. Opt. Mater.* **4**, 1482–1486 (2016).

S3. Al Saghir, K. *et al.* Transparency through Structural Disorder: A New Concept for Innovative Transparent Ceramics. *Chem. Mater.* **27**, 508–514 (2015).

S4. Allix, M. *et al.* Highly Transparent $BaAl_4O_7$ Polycrystalline Ceramic Obtained by Full Crystallization from Glass. *Adv. Mater.* **24**, 5570–5575 (2012).

S5. Boyer, M. *et al.* Enhanced Transparency through Second Phase Crystallization in $BaAl_4O_7$ Scintillating Ceramics. *Cryst. Growth Des.* **16**, 386–395 (2016).

S6. Alahraché, S. *et al.* Perfectly Transparent $Sr_3Al_2O_6$ Polycrystalline Ceramic Elaborated from Glass Crystallization. *Chem. Mater.* **25**, 4017–4024 (2013).

S7. Zheng, G. *et al.* Glass-Crystallized Luminescence Translucent Ceramics toward High-Performance Broadband NIR LEDs. *Adv. Sci.* **9**, 2105713 (2022).

S8. Irifune, T. *et al.* Pressure-induced nano-crystallization of silicate garnets from glass. *Nat. Commun.* **7**, 13753 (2016).

S9. Mei, L., He, G., Wang, L.-L., Liu, G.-H. & Li, J.-T. Fabrication of transparent $LaAlO_3$/t-ZrO_2 nanoceramics through controlled amorphous crystallization. *J. Eur. Ceram. Soc.* **31**, 1603–1609 (2011).

S10. Fu, J. *et al.* Ce^{3+}:$Lu_3Al_5O_{12}$–Al_2O_3 optical nanoceramic scintillators elaborated via a low-temperature glass crystallization route. *J. Adv. Ceram.* **12**, 268–278 (2023).

S11. Fu, J. *et al.* Transparent perovskite-based nanoceramics elaborated from full glass crystallization. *Nanoscale* **17**, 17127–17136 (2025).

S12. Ma, X. *et al.* Pressureless glass crystallization of transparent yttrium aluminum garnet-based nanoceramics. *Nat. Commun.* **9**, 1175 (2018).

S13. Tokariev, O., Steinbrech, R. W., Schnetter, L. & Malzbender, J. Micro- and macro-mechanical testing of transparent MgAl2O4 spinel. *J. Mater. Sci.* **47**, 4821–4826 (2012).

S14. Zhou, Z. *et al.* Characterization and evaluation on mechanical property of $Mg_{0.27}Al_{2.58}O_{3.73}N_{0.27}$ transparent ceramic. *J. Inorg. Mater.* **33**, 1006 (2018).

S15. Chen, P. *et al.* Fabrication, microstructure, and properties of 8 mol% yttria-stabilized zirconia (8YSZ) transparent ceramics. *J. Adv. Ceram.* **11**, 1153–1162 (2022).

S16. Wang, H. M. *et al.* Unique mechanical properties of nano-grained YAG transparent ceramics compared with coarse-grained partners. *Mater. Des.* **105**, 9–15 (2016).

S17. Guo, H. *et al.* Effects of AlN content on mechanical and optical properties of AlON transparent ceramics. *Ceram. Int.* **46**, 16677–16683 (2020).

S18. Zhang, L., Feng, J. & Pan, W. Vacuum sintering of transparent Cr:Y_2O_3 ceramics. *Ceram. Int.* **41**, 8755–8760 (2015).

S19. Wang, B. *et al.* A novel durable spinel-type $ZnGa_2O_4$ transparent ceramic with wide transmission range. *Scr. Mater.* **205**, 114186 (2021).

S20. Akchurin, M. Sh. *et al.* CaF_2:Yb laser ceramics. *Opt. Mater.* **35**, 444–450 (2013).

S21. Li, J. *et al.* Fabrication, microstructure and properties of highly transparent Nd:YAG laser ceramics. *Opt. Mater.* **31**, 6–17 (2008).

S22. Tang, H. *et al.* Toughening oxide glasses through paracrystallization. *Nat. Mater.* **22**, 1189–1195 (2023).
