# Supplementary material for: Breaking the grain size limit of glass-crystallized transparent nanoceramics via multi-phase interface locking
Source: Nat Commun. 2026 Aug 15;17:9825. doi: 10.1038/s41467-026-76601-5 (PMC13575170; doi:10.1038/s41467-026-76601-5)
Supplement: Supplementary file 3 [file 41467_2026_76601_MOESM3_ESM.pdf]

nature portfolio

Peer Review File

Breaking the grain size limit of glass-crystallized transparent nanoceramics via multi-phase interface locking

Corresponding Author: Professor Jianqiang Li

This file contains all reviewer reports in order by version, followed by all author rebuttals in order by version.

Version 0:

Reviewer comments:

Reviewer #1

(Remarks to the Author)
This work reports the fabrication of fully crystallized transparent ceramics with a claimed grain size of 16 nm through crystallization of a multicomponent glass precursor. The method leverages the advantages of glass crystallization, thereby avoiding the challenges typically associated with conventional sintering processes for transparent ceramics. Owing to the multicomponent glass precursor, complex interfacial pinning effects enabled the formation of the multiphase ceramic with high optical transparency and enhanced mechanical properties under relatively mild conditions, achieving a record-small grain size. This work provides valuable insights into the design of transparent ceramics. The emergence of such multiphase transparent ceramics may open up new possibilities for developing multifunctional integrated transparent materials through synergistic interactions between different crystalline phases. The manuscript is considered suitable for publication after addressing the following points:

1. Achieving such fine grain sizes (~16 nm) is generally challenging through conventional ceramic processing routes. Beyond the reported high optical transparency and moderately improved mechanical properties, it would be meaningful to explore whether this ultra-fine microstructure also leads to other enhanced functional properties?

2. It is standard practice in nanoindentation testing to perform a minimum of five measurements to ensure statistical reliability. Therefore, error bars should be added to the reported values of hardness and Young's modulus to represent the data scatter.

3. In transparent ceramics, the presence of multiple crystalline phases conventionally leads to increased scattering losses at phase boundaries, making high phase purity a common prerequisite for optimal transparency. This appears to contradict the entropy-engineered fabrication strategy, which often requires multiple components. Could the authors clarify this apparent contradiction?

4. In this work, the key factor enabling suppression of grain growth is the interfacial pinning effect resulting from the simultaneous crystallization of multiple phases. A critical question therefore arises: how universal is this mechanism? What are the prospects for extending this glass-crystallization approach to other multicomponent systems for the development of transparent ceramics?

Reviewer #2

(Remarks to the Author)
The evaluation methods for the in-line transmittance and mechanical properties, the most important results of the present study, are still unclear. These should be carefully discussed to properly evaluate the significance of this work. The accuracy of the transparency measurement could significantly affect the conclusions of this paper.

Figure 3 and Supplementary Fig. 2 contain photographs demonstrating the samples' translucency. However, in both cases, the sample was placed directly on a printed sheet, and these photographs alone do not prove the high in-line transmittance. If the sample and the measurement position of the transmitted light are close to each other, scattered light is likely to be counted as transmitted light, resulting in an overestimate of the in-line transmittance. The distances between the light source

and the sample, as well as between the sample and the device for the transmitted light measurement, must be clearly presented. The transmitted light measurement method (an integrating sphere?) must be also described.

The determination of Young's modulus using a nanoindenter and the measurement method for KIC should also be described in detail. For the latter in particular, the SEPB method would be more appropriate than Vickers indentation. The fracture toughness values would be determined by IF method in the present study, but it should be noted that the IF method may significantly overestimate KIC values compared to the SEPB method.

Page 6, Lines 142 and 157
Isothermal holding at 1100°C for 2 hours is effective in enhancing the crystallization, but there is little evidence that isothermal holding at temperatures lower than 1100C leads to complete crystallization. Even for the samples isothermally held at 1100C, it is unconvincing to conclude "complete" crystallization (i.e., the absence of any amorphous phase) based solely on DSC or XRD results. To demonstrate the absence of an amorphous phase, for example, an electron-beam diffraction pattern from a wide area in a TEM sample must be obtained, demonstrating the absence of a halo from amorphous phase.

Page 9, Line 205
The STEM-HAADF image in Figure 2 shows a sample with extremely fine and complex crystallization. Although the sample crystallized at 1100C for 2 hours is undoubtedly composed of very fine crystals, the grain size of 16 nm (mentioned in the conclusion) is questionable. Figures 2(b) and (c) show La(TiAl)O phases several tens of nanometers in size.

Reviewer #3

(Remarks to the Author)
In this work, the authors developed a novel material with high mechanical properties, a high refractive index, but a low vd, showing high potential in future optical devices. The manuscript is well - organized, but some comments still have to be made:
1.In Fig. 1g, the sharp XRD peaks are not solid evidence for "complete crystallization". Since the authors use the glass crystallization method to prepare transparent ceramics, it is very important to confirm the full crystallization of the glasses. If not, it is just a common method to make transparent glass - ceramic, and nano - sized crystals in the glassy phase have been widely reported. In addition, some glass - ceramics with high crystallinity, even over 90%, have also been reported.
2.It is suggested to add the scale bar for the samples in Fig 3a. And, usually the transmittance at 550 nm is used to evaluated for the transparency in the visible light range.
3.The test method is significantly affect the results for the mechanical properties. In this work, the hardness was tested under a load of 4.9 N for 10 s. The applied load is smaller than the commonly used value. I wonder if it would affect the final results? In addition, it is suggestd to compare the modulus and KIC of nano-ceramics in this work to the reported transparent ceramics measured with the same method.
4.Please clarify the crystallization and growth mechanism of the obtained nano-ceramics to highlight the novelty of this work. Regard to the title of this manuscript, glass-crystallized with nanosized crystals and ultra-fine size distribution is not limited. And how the multi-phase interface locking favor to obtain transparent nanocermaics?

Reviewer #4

(Remarks to the Author)
Reviewer Report (for Nature Communications)

Summary
In this manuscript, the authors report the fabrication of highly transparent multiphase nanoceramics derived from a high-entropy glass precursor, achieving an exceptionally small average grain size (~16 nm) despite the coexistence of three crystalline phases. By combining detailed structural characterization with optical and mechanical measurements, the authors claim that entropy-assisted glass crystallization suppresses grain growth and enables an unusual combination of multiphase coexistence and high transparency.
The experimental work is carefully conducted, and the dataset is extensive. The reported grain size is indeed among the smallest achieved for bulk transparent ceramics prepared via glass crystallization. However, while the technical achievement is impressive, the manuscript in its current form lacks a sufficiently clear conceptual advance to fully justify publication in Nature Communications. Several key claims—particularly regarding the role of entropy—require clearer justification and sharper framing.

Major Comments
1. Insufficient clarity of the central conceptual advance
At present, the manuscript presents multiple strong results (ultrafine grain size, multiphase coexistence, high transparency, improved mechanical properties), but these are treated largely on an equal footing. As a consequence, the core message of the paper is diluted.
For a broad-scope journal such as Nature Communications, the manuscript must articulate a single, clearly defined conceptual advance. It is currently unclear whether the main contribution is:
• the achievement of a record-small grain size via glass crystallization,
• the demonstration of high optical transparency in a multiphase ceramic,
• or the proposal of entropy as a governing parameter for crystallization kinetics.
The authors are strongly encouraged to reformulate the manuscript around one dominant claim, with the remaining results

positioned as supporting evidence rather than parallel achievements.

2. Ambiguity in the role of entropy as a controlling parameter
The manuscript repeatedly emphasizes the importance of configurational entropy in suppressing grain growth. However, the presented evidence does not yet convincingly demonstrate that entropy is the primary controlling factor, rather than a correlated descriptor.
A critical reader may reasonably ask:
Is entropy itself responsible for the suppressed grain growth, or are kinetic factors (e.g., reduced cation mobility, multiphase boundary pinning) the dominant mechanism?
To address this concern, the authors should consider one or more of the following:
• Comparative experiments using lower-entropy compositions with otherwise similar chemistry,
• A more explicit kinetic or thermodynamic framework explaining how entropy quantitatively affects crystallization and grain growth,
• Or a reframing of entropy as a contributing, rather than governing, factor.
Without such clarification, the entropy-based interpretation risks appearing post hoc.

3. Contextualization of the reported grain size
The reported average grain size of ~16 nm is clearly remarkable. However, its significance is not sufficiently conveyed to non-specialist readers.
The authors should provide a systematic comparison with prior reports on transparent ceramics derived from glass crystallization, ideally in the form of a table or summary figure. This comparison should include:
• Material systems,
• Processing routes,
• Achieved grain sizes,
• Optical transparency (where available).
Such contextualization would substantially strengthen the manuscript and allow editors and readers to immediately appreciate the advance.

4. Multiphase coexistence versus transparency: missed opportunity
One of the most intriguing aspects of this work is the coexistence of three crystalline phases without significant loss of optical transparency. This result runs counter to conventional expectations in ceramic optics.
However, this apparent contradiction is not sufficiently emphasized or conceptually exploited. The authors should explicitly address why multiphase coexistence, typically detrimental to transparency, does not degrade optical performance in this case, and how the ultrafine grain size fundamentally alters this paradigm.
Framing this point more explicitly could significantly enhance the manuscript's conceptual impact.

5. Overextension of the results
The manuscript covers a wide range of properties (structural, optical, mechanical, thermodynamic), which risks overextending the narrative. Some sections read as descriptive rather than hypothesis-driven.
For improved focus, the authors should consider prioritizing the results most directly supporting the central claim, while relocating secondary data to the Supplementary Information.

Minor Comments
1. The Introduction is overly long and at times resembles a review article. Streamlining would improve readability.
2. Claims of first"or"unprecedented"should be supported by explicit comparisons or softened where appropriate.
3. The limitations of Scherrer-based grain size estimation should be discussed more explicitly, particularly in comparison with STEM-derived values.
4. Several causal statements rely on speculative phrasing (e.g., "may be attributed to"). Where possible, these should be strengthened or qualified more precisely.

Recommendation
Major Revision
The manuscript reports a technically impressive and potentially impactful result. However, substantial revision is required to sharpen the conceptual focus, clarify the role of entropy, and strengthen the framing for a broad materials science audience.

Version 1:

Reviewer comments:

Reviewer #1

(Remarks to the Author)
All the concerns of this reviewer have been properly and adequately addressed, and the work may now be accepted for publication.

Reviewer #2

(Remarks to the Author)

The authors have added substantial results and discussions, particularly regarding the evaluation of optical and mechanical properties.

Reviewer #4

(Remarks to the Author)
The revised manuscript has substantially improved in focus and now presents a clearer central message centered on multi-phase interface locking and sub-20 nm grain-size control. The role of entropy has been appropriately deemphasized, and the discussion of transparency in a multiphase nanoceramic is much clearer. I consider the revision largely responsive to my major concerns.

Before final acceptance, I recommend minor textual revisions:
(i) ensure consistency between the response letter and manuscript regarding the location of the comparison table; if the table is central to contextualizing the grain-size advance, it should preferably appear in the main text rather than only in the Supplementary Information;
(ii) soften absolute wording such as “record-fine” unless fully supported by the main-text comparison;
(iii) moderate remaining strong causal language in the Abbe-number and mechanical-property discussions, where the mechanistic evidence is less direct than for grain-size suppression.

With these minor changes, I believe the manuscript would satisfactorily address my previous concerns.

Open Access This Peer Review File is licensed under a Creative Commons Attribution 4.0 International License, which permits use, sharing, adaptation, distribution and reproduction in any medium or format, as long as you give appropriate credit to the original author(s) and the source, provide a link to the Creative Commons license, and indicate if changes were made.
In cases where reviewers are anonymous, credit should be given to 'Anonymous Referee' and the source.
The images or other third party material in this Peer Review File are included in the article’s Creative Commons license, unless indicated otherwise in a credit line to the material. If material is not included in the article’s Creative Commons license and your intended use is not permitted by statutory regulation or exceeds the permitted use, you will need to obtain permission directly from the copyright holder.
To view a copy of this license, visit https://creativecommons.org/licenses/by/4.0/

Reviewer #1

Opening comments: *This work reports the fabrication of fully crystallized transparent ceramics with a claimed grain size of 16 nm through crystallization of a multicomponent glass precursor. The method leverages the advantages of glass crystallization, thereby avoiding the challenges typically associated with conventional sintering processes for transparent ceramics. Owing to the multicomponent glass precursor, complex interfacial pinning effects enabled the formation of the multiphase ceramic with high optical transparency and enhanced mechanical properties under relatively mild conditions, achieving a record-small grain size. This work provides valuable insights into the design of transparent ceramics. The emergence of such multiphase transparent ceramics may open up new possibilities for developing multifunctional integrated transparent materials through synergistic interactions between different crystalline phases. The manuscript is considered suitable for publication after addressing the following points.*

Response: We sincerely appreciate reviewer #1's positive evaluation and constructive feedback on our manuscript. We have carefully considered all comments and have addressed them point-by-point in the revised manuscript.

Comment #1: *Achieving such fine grain sizes (~16 nm) is generally challenging through conventional ceramic processing routes. Beyond the reported high optical transparency and moderately improved mechanical properties, it would be meaningful to explore whether this ultra-fine microstructure also leads to other enhanced functional properties?*

Response: Thank you very much for your insightful comment, with which we fully agree. Exploring additional functional properties of this nanocrystalline material would indeed be valuable, and we appreciate the opportunity to more clearly articulate the significance of what we have already achieved in this work.

The central challenge we set out to address is not simply the fabrication of fine-grained transparent ceramics, but rather a fundamental contradiction in ceramic optics: multiphase coexistence is conventionally considered incompatible with high transparency because each phase boundary acts as a scattering center. This limitation has historically forced transparent ceramic design toward high phase purity, severely restricting the compositional space. Our work overcomes this long-standing limitation by demonstrating that when grain sizes are reduced to the deep sub-wavelength scale (<20 nm) via multi-phase interface locking, the scattering contribution from phase boundaries becomes negligible regardless of their number or refractive index mismatch. The key insight is that transparency is governed by the size of the scattering features relative to the wavelength, not by phase purity per se. This finding fundamentally shifts the design paradigm: transparent ceramics no longer need to be single-phase or even dual-phase; complex multiphase assemblages become viable

as long as the microstructure is sufficiently refined.

The multi-phase interface locking strategy not only enables transparency in multiphase systems but also enhances mechanical performance. The conceptual advance, therefore, lies in proving that phase purity is not a prerequisite for transparency; rather, the critical parameter is the microstructural scale. This insight opens new avenues for designing transparent materials with intentionally complex phase compositions, which can be tailored for multifunctional applications. While systematic investigation of other functional properties (e.g., thermal conductivity, electrical behavior, or superplasticity) is beyond the scope of this work, the nanocrystalline multiphase architecture established here provides a promising platform for such future explorations.We have reframed the discussion in the revised manuscript to emphasize this conceptual breakthrough, and we hope this focus appropriately highlights the significance of our work.

Comment #2: *It is standard practice in nanoindentation testing to perform a minimum of five measurements to ensure statistical reliability. Therefore, error bars should be added to the reported values of hardness and Young's modulus to represent the data scatter.*

Response: Thank you very much for your comment. We shall follow your suggestion and add error bars for the data of hardness and Young's modulus in Figure R1-1.

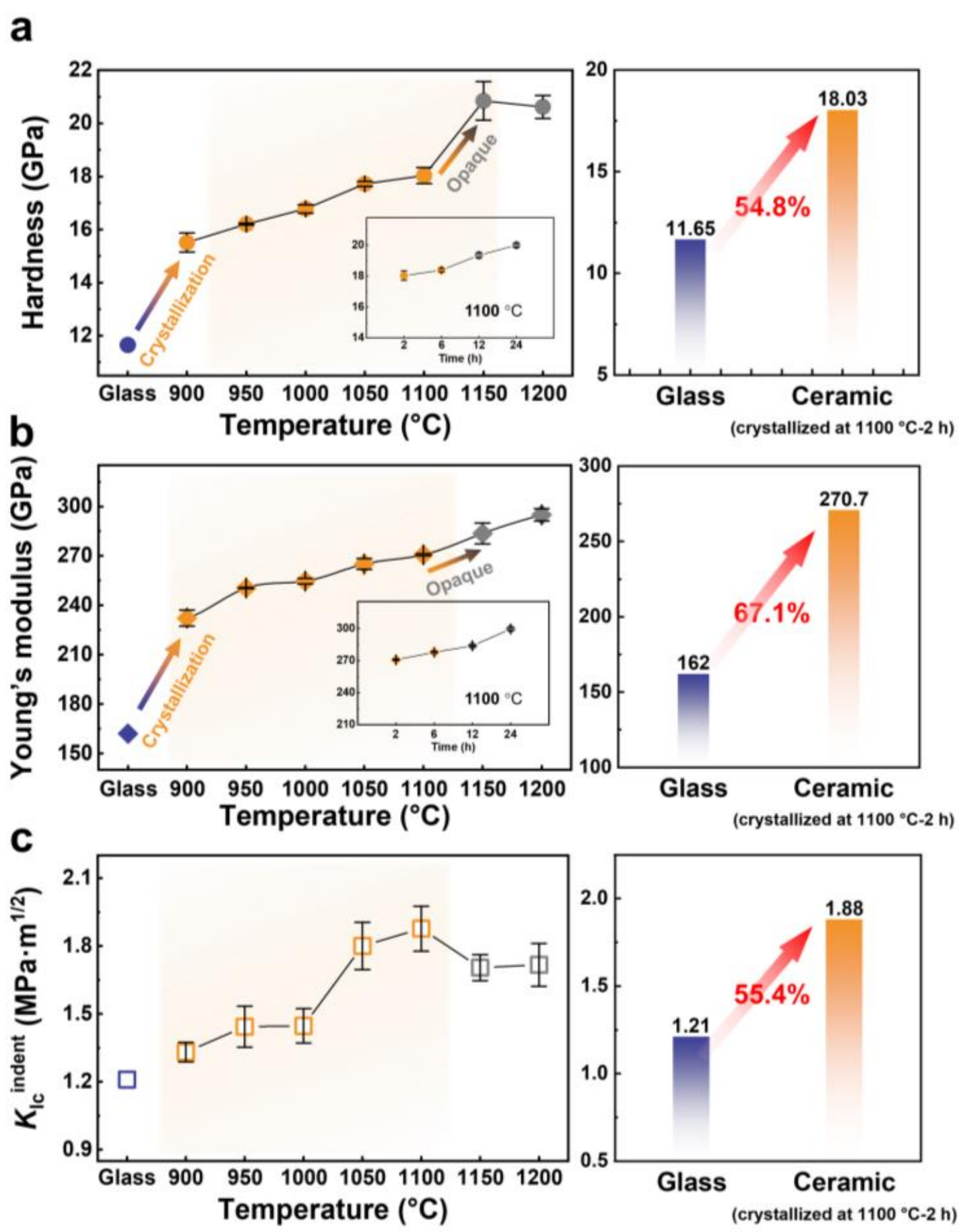

Figure R1-1 Mechanical properties of multi-phase nanocrystalline ceramics. a,b, Hardness and Young's modulus of samples crystallized from LYTZA glass at 900–1200 °C for 2 h (The inset is samples crystallized from LYTZA glass at 1100 °C for 2–24 h). **c,** Indentation fracture toughness (K_{Ic}^{indent}) of samples crystallized from LYTZA glass at 900–1200 °C for 2 h.

Changes: We have added error bars for the data of hardness and Young's modulus and updated Figure 4 in the revised manuscript.

Comment 3: *In transparent ceramics, the presence of multiple crystalline phases conventionally leads to increased scattering losses at phase boundaries, making high phase purity a common prerequisite for optimal transparency. This appears to contradict the entropy-engineered fabrication strategy, which often requires multiple components. Could the authors clarify this apparent contradiction?*

Response: We thank the reviewer for raising this insightful point regarding the apparent contradiction between multiphase composition and optical transparency. The concern is well-founded: in conventional ceramics, scattering at phase boundaries due to refractive index mismatch is indeed a major barrier to transparency, making phase purity a common design goal. In this work, the key to resolving this contradiction lies not in avoiding multiple phases, but in exploiting multi-phase interface locking to render the scattering centers (grain sizes of each phase) smaller than the wavelength of visible light.

Specifically, the multi-component glass precursor is designed to provide abundant nucleation sites, promoting the simultaneous and rapid precipitation of three distinct crystalline phases. Crucially, once this multiphase nanostructure is formed, grain growth becomes kinetically constrained: it requires not only phase-boundary migration but also long-range atomic transport from source to sink of the same phase across dissimilar grains, a process far slower than the local atomic rearrangements in single-phase systems. This sluggish growth kinetics effectively suppresses grain coarsening, locking in an exceptionally fine nanocrystalline structure with an average grain size below 20 nm. At this scale (≈1/30 of the wavelength of visible light), the scattering cross-section of phase boundaries becomes negligible regardless of their number or refractive index mismatch. Therefore, our approach does not contradict the conventional principle of minimizing scattering; rather, it achieves the same goal through a different mechanism: size-dominated transparency enabled by multi-phase interface locking.

Changes: We have included discussion on this topic in the revised manuscript.

Comment 4: *In this work, the key factor enabling suppression of grain growth is the interfacial*

pinning effect resulting from the simultaneous crystallization of multiple phases. A critical question therefore arises: how universal is this mechanism? What are the prospects for extending this glass-crystallization approach to other multicomponent systems for the development of transparent ceramics?

Response: We thank the reviewer for this critical question. The universal principle demonstrated in this work is that optical transparency in ceramics is primarily governed by grain size rather than by the presence of any specific crystalline phase. As long as the grain size can be confined to the deep sub-wavelength nanoscale (e.g., <20 nm as achieved here) via multi-phase interface locking, high transparency can be realized even in multiphase systems. Therefore, the glass-crystallization route reported here holds promising potential for extension to other multi-component systems. Guided by this principle, we are now working to extend this approach to other multicomponent glass systems for the development of transparent ceramics with diverse functional properties. As a preliminary demonstration, we have crystallized samples of the 31.6RO-4.1Y_2O_3-23.7TiO_2-7.2ZrO_2-33.2Al_2O_3 system (R = Ba, Ca) at 850–1050 °C for 2 h (Figure R1-2). The crystallized samples exhibit at least three crystalline phases, show high transmittance of approximately 85% in the near- to mid-infrared region comparable to that of the glass precursors, and display significantly enhanced mechanical properties compared to the glasses. These results further highlight the universality of our approach.

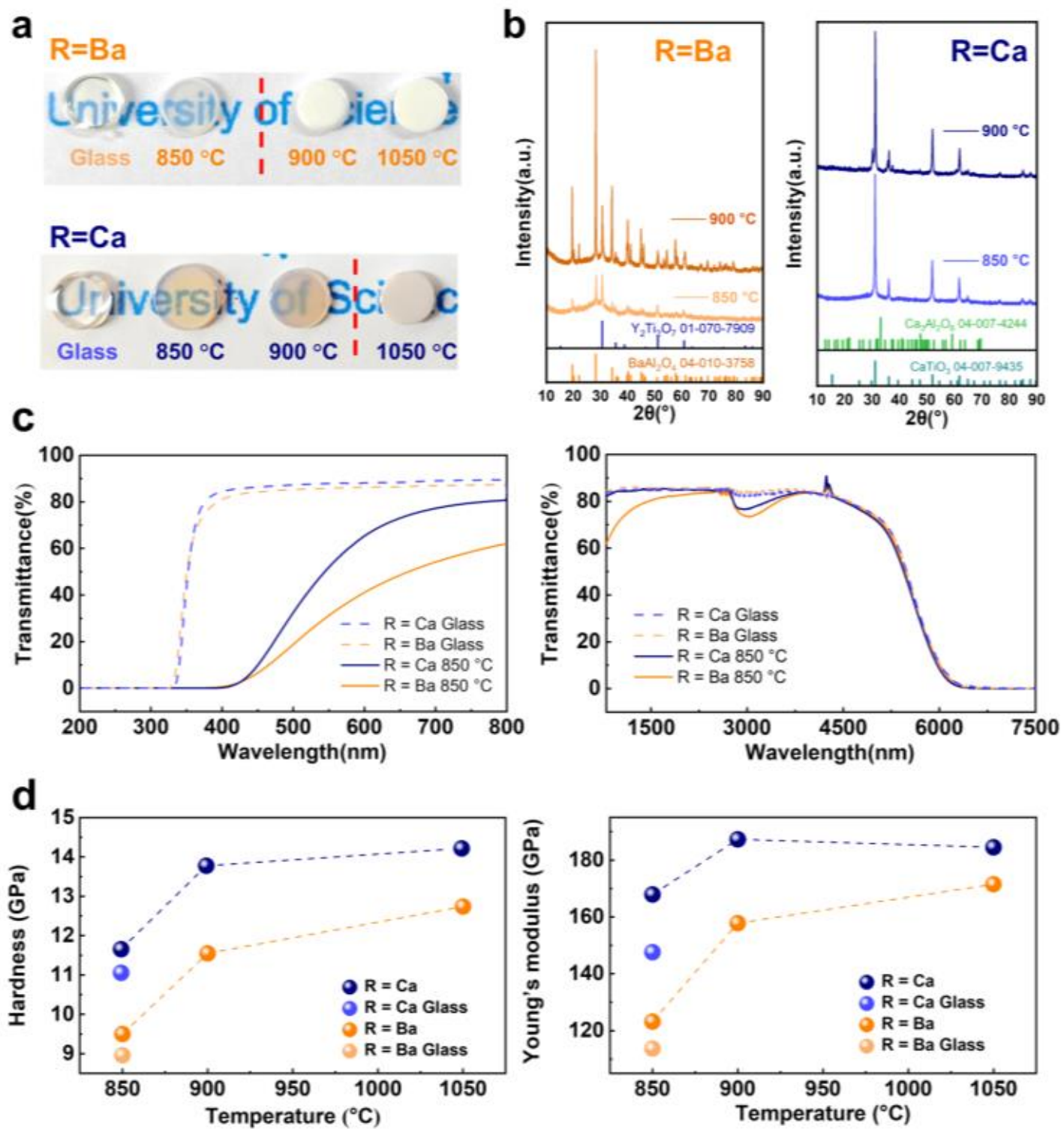

Figure R1-2 Multi-phase nanocrystalline ceramics crystallized from other multi-component glass systems. a, Photographs of samples crystallized from BYTZA and CYTZA glasses at 850–1050 °C for 2 h. **b,** XRD patterns of samples crystallized from BYTZA and CYTZA glasses at

850 °C and 900 °C for 2 h. **c,** In-line transmittance spectra (1 mm thickness) of samples crystallized from BYTZA and CYTZA glasses at 850 °C and 900 °C for 2 h. **d,** Hardness and Young's modulus of samples crystallized from BYTZA and CYTZA glasses at 850 °C and 900 °C for 2 h.

We would like to express our gratitude once again for your time and effort in reviewing our manuscript.

Reviewer #2

Opening comments: *The evaluation methods for the in-line transmittance and mechanical properties, the most important results of the present study, are still unclear. These should be carefully discussed to properly evaluate the significance of this work. The accuracy of the transparency measurement could significantly affect the conclusions of this paper.*

Response: We sincerely thank reviewer #2 for raising this important point regarding the clarity of our evaluation methods. We fully agree that a detailed description and discussion of the characterization techniques, particularly for in-line transmittance and mechanical properties, are essential for properly assessing the significance and reliability of our findings.

Comment 1: *Figure 3 and Supplementary Fig. 2 contain photographs demonstrating the samples' translucency. However, in both cases, the sample was placed directly on a printed sheet, and these photographs alone do not prove the high in-line transmittance. If the sample and the measurement position of the transmitted light are close to each other, scattered light is likely to be counted as transmitted light, resulting in an overestimate of the in-line transmittance. The distances between the light source and the sample, as well as between the sample and the device for the transmitted light measurement, must be clearly presented. The transmitted light measurement method (an integrating sphere?) must be also described.*

Response: We thank the reviewer for raising these critical points. We have made the following improvements in the revised manuscript.

To more rigorously demonstrate transparency, we have re-taken all relevant photographs (Fig. 3a) by positioning the samples on a polycarbonate board placed 2 cm above the background, following the method described in previous studies (Kim, B.-N. et al., *Acta Materialia*, 57, 1319–1326, 2009; Lee, S.-H. et al., *Journal of the American Ceramic Society*, 89, 1945–1950, 2006). For comparison, we also include photographs taken with the sample placed directly on the background, along with images of the underlying text captured through the elevated sample using a telephoto lens (Supplementary Fig. 3; Figure R2-1 in this response).

For the transmitted light measurement method, we chose to measure the in-line transmittance instead of the total transmittance (which would require an integrating sphere) because the former is critical for evaluating the "see-through clarity" of transparent ceramics, directly reflecting the intensity loss due to scattering and absorption. While an integrating sphere would include forward-scattered light and might yield a higher numerical value, the in-line transmittance is a more relevant metric for many target applications, such as optical windows or transparent armor.

The in-line transmittance of the samples (each 1.0 mm thick) was measured across the ultraviolet–visible–near-infrared (UV–Vis–NIR) region (200–2500 nm) using a spectrophotometer (Cary 5000, Agilent Technologies, America; Figure R2-2) in its standard transmission configuration. A dual-beam optical arrangement with 1 mm apertures on both the reference and sample beams was employed, and the sample was precisely secured in the compartment with air as the baseline. In this calibrated setup, the sample was positioned at the standard height (Z-axis ≈ 20 mm) within the sample chamber, where the factory-calibrated light source and detector are fixed relative to each other. This geometry ensures that only directly transmitted light within a small solid angle is collected, minimizing scattered light contributions. This method is a well-established and reliable approach for quantifying the transparency of ceramics (Yiquan Wu et al., *International Materials Reviews*, 68, 648–676, 2023). These details have been added to the optical property measurement section of the "Methods".

We believe these clarifications and additions allow for a more accurate and rigorous presentation of our optical characterization results.

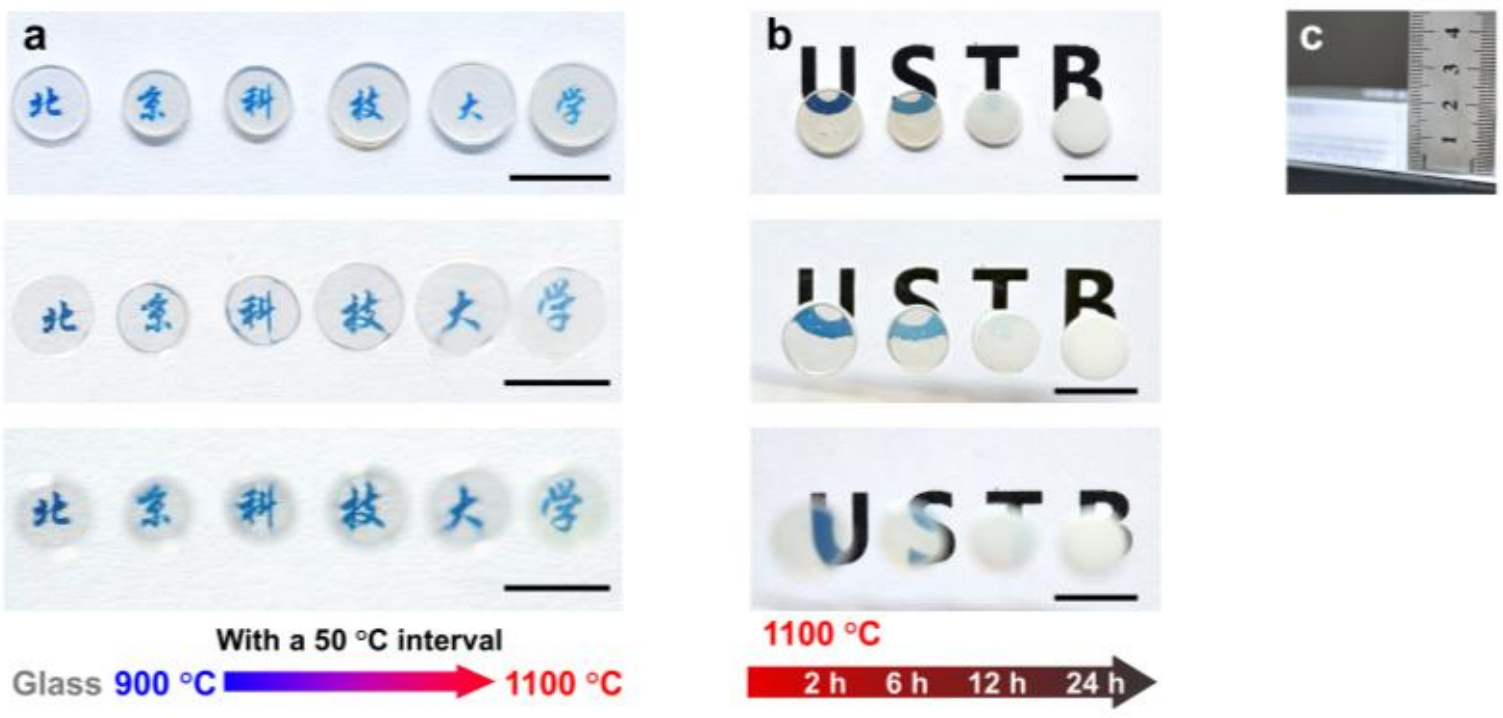

Figure R2-1 Sample photographs and imaging setup. a, LYTZA glass samples crystallized at 900–1100 °C for 2 h. For each sample, three photographs are shown from top to bottom: placed directly on the background, placed on a polycarbonate board 2 cm above the background using a standard lens, and using a telephoto lens. **b,** LYTZA glass samples crystallized at 1100 °C for 2–24 h, arranged in the same order as in **a**. **c,** Photograph of the polycarbonate board used for sample elevation. Scale bars in **a** and **b** are 5 mm.

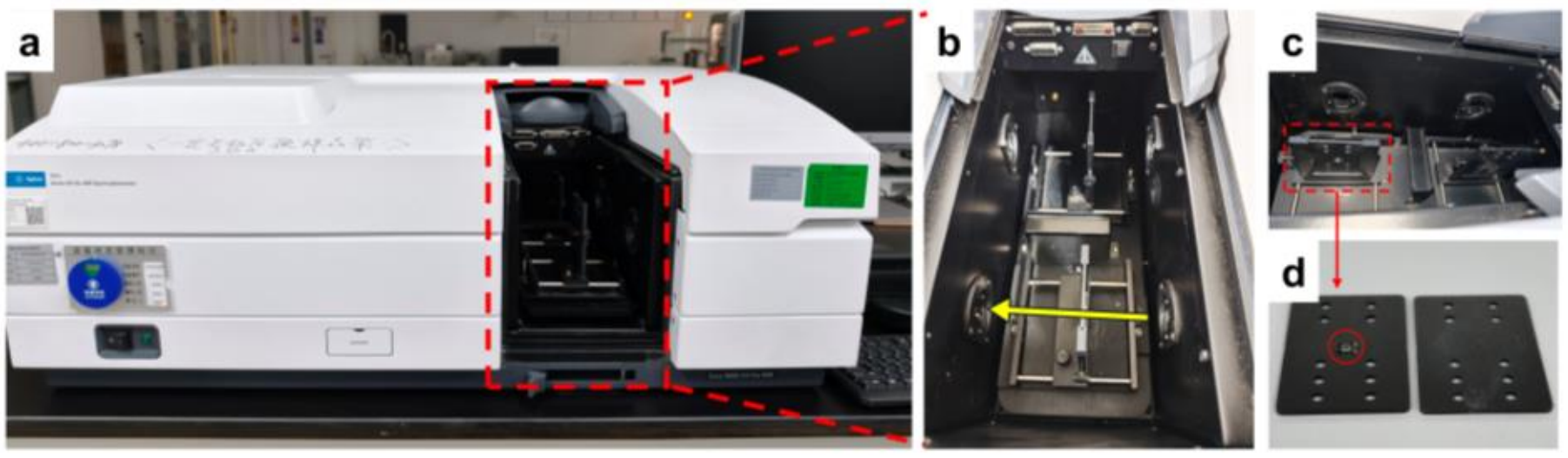

Figure R2-2 In-line transmittance measurement setup using a spectrophotometer. a,

Photograph of the spectrophotometer. **b,** Front view of the sample holder inside the instrument. **c,** Side view of the sample holder. **d,** Photograph showing a sample fixed on a quartz glass plate.

Changes: We have re-taken the sample photographs (Fig. 3a and Supplementary Fig. 3) and added a detailed description of the in-line transmittance measurement method to the "Methods" section.

Comment 2: *The determination of Young's modulus using a nanoindenter and the measurement method for KIC should also be described in detail. For the latter in particular, the SEPB method would be more appropriate than Vickers indentation. The fracture toughness values would be determined by IF method in the present study, but it should be noted that the IF method may significantly overestimate KIC values compared to the SEPB method.*

Response: We thank the reviewer for the important comments regarding the characterization methods for mechanical properties. We have followed the suggestions and expanded the "Methods" section to include specific parameters for both nanoindentation and fracture toughness testing. The revised description now includes the detailed formulas for hardness and Young's modulus based on the Oliver–Pharr model, as well as the empirical relation used for indentation fracture toughness (K_{Ic}^{indent}) calculation. The revised content is as follows:

"The hardness and Young's modulus of the samples were measured using a nanoindenter (G200, Keysight Technologies, USA) with a penetration depth of 2000 nm. The hardness and Young's modulus of the samples were derived based on the Oliver–Pharr model. The hardness (H_V) was calculated using the following formula:

$$H_v = \frac{P}{A} \tag{6}$$

where P is the indentation load and A is the projected contact area under the indenter.
Young's modulus (E) was obtained from the relation:

$$E = \frac{\sqrt{\pi}}{2\beta}\frac{S}{\sqrt{A}} \tag{7}$$

where S is the elastic contact stiffness and β is a correction factor (approximately 1.034 for a Berkovich indenter). The contact stiffness S was determined as:

$$S = \lambda m(h_{max} - h_f)^{m-1} \tag{8}$$

where λ and m are fitting parameters, h_{max} is the maximum indentation depth, and h_f is the residual depth after unloading. At least five indentations were performed on each sample using a diamond (Berkovich) indenter, and the average value was reported.
The fracture toughness was evaluated using a Vickers hardness tester (HMAS-D1000Z, Shanghai, China) under a load of 4.9 N with a dwell time of 10 s. During the test, a well-defined indentation and associated cracks are produced on the sample surface. By measuring the crack lengths and combining them with the corresponding load, hardness, and Young's modulus, the indentation

fracture toughness (K_{Ic}^{indent}) was calculated using the following empirical relation[69]:

$$K_{Ic}^{indent} = 0.016\left(\frac{E}{H_V}\right)^{0.5} \frac{P}{c^{1.5}} = 0.23(EP)^{0.5} \frac{a}{c^{1.5}} \tag{9}$$

where E is the Young's modulus, P is the applied load, c is the average crack length from the indentation centre to the crack tip, and a is the half-diagonal length of the Vickers impression. To enhance measurement accuracy, at least ten indentations were made in different areas of each sample using a diamond indenter."

We agree that the Single Edge Precracked Beam (SEPB) method is generally preferred for fracture toughness measurement. However, due to the small size (3–5 mm) of our samples, which were prepared via a containerless solidification technique, they do not meet the standard size requirements for the SEPB test. Therefore, the IF (Indentation Fracture) method was employed as a practical alternative. Generally, the accuracy of fracture toughness measurements using the indentation method is limited, with a systematic bias: the measured toughness of brittle materials with low fracture toughness is often overestimated, whereas that of high-toughness brittle materials is frequently underestimated (Sant'Ana Gallo, L. et al., *Ceramics International*, 48, 9906–9917, 2022; Dériano, S. et al., *Journal of Non-Crystalline Solids*, 344, 44–50, 2004; Sellappan, P. et al., *Acta Materialia*, 61, 5949–5965, 2013). Nevertheless, for our material system, where the measured fracture toughness falls within a moderate range (1.33–1.88 $MPa\cdot m^{1/2}$), which is less susceptible to the most significant overestimation or underestimation biases. Moreover, the primary goal here is comparative assessment rather than absolute value determination. Therefore, the IF method remains a valid and practical choice for this work.

To prevent any misinterpretation, we have added a clarifying note in the revised manuscript regarding the fracture toughness measurement. Specifically, we denote the values as K_{Ic}^{indent} to indicate that they were obtained via the IF method.

Changes: We have added detailed descriptions of the above characterization methods and labelled the fracture toughness using indentation technique as K_{Ic}^{indent}.

Comment 3: *Page 6, Lines 142 and 157: Isothermal holding at 1100°C for 2 hours is effective in enhancing the crystallization, but there is little evidence that isothermal holding at temperatures lower than 1100°C leads to complete crystallization. Even for the samples isothermally held at 1100°C, it is unconvincing to conclude "complete" crystallization (i.e., the absence of any amorphous phase) based solely on DSC or XRD results. To demonstrate the absence of an amorphous phase, for example, an electron-beam diffraction pattern from a wide area in a TEM sample must be obtained, demonstrating the absence of a halo from amorphous phase.*

Response: We thank the reviewer for raising this critical point. We fully agree that the claim of "complete crystallization" requires stronger evidence than XRD and DSC results alone. The Rietveld refinement results further support this conclusion: the phase ratio of $La(Al_{1/3}Ti_{2/3})O_3$ to $Y_2(Zr_{0.6}Ti_{0.4})_2O_7$ was determined to be 85.3% to 14.7%, closely matching the nominal composition ratio of 88.6% to 11.4%. This close agreement indicates that virtually all La_2O_3, Y_2O_3, TiO_2, and ZrO_2 present in the glass are consumed to form the respective nanocrystalline phases.

In the manuscript, in addition to XRD and DSC analyses, we have also presented density as a complementary indicator. As shown in Fig. 1b, the density stabilized at 5.139 g cm^{-3} after crystallization at 1100 °C for 2 hours. This represents an increase of 12.57% relative to the glass precursor (4.565 g cm^{-3}), 2.7% relative to the sample crystallized at 900 °C for 2 hours (5.004 g cm^{-3}), and only about 0.14% difference relative to the samples crystallized at 1150 °C (5.132 g cm^{-3}) and 1200 °C (5.137 g cm^{-3}) for 2 hours, with no significant change (within 0.07%) observed upon further extending the holding time (5.135–5.142 g cm^{-3}). Based on the nominal composition, the theoretical density of the ceramic was calculated to be 5.142 g cm^{-3}, which is in good agreement with the measured density of 5.139 g cm^{-3}. This evolution in density, together with the consistency between the measured and theoretical values, provides further evidence that the crystallization process has reached completion.

Furthermore, directly addressing the reviewer's suggestion, we have now supplemented the revised manuscript with electron diffraction patterns obtained from wide areas in TEM (Supplementary Fig. 2; Figure R2-3 in this response). These patterns exhibit sharp diffraction spots with no diffuse halos characteristic of an amorphous phase, providing direct evidence for the absence of a residual glassy phase.

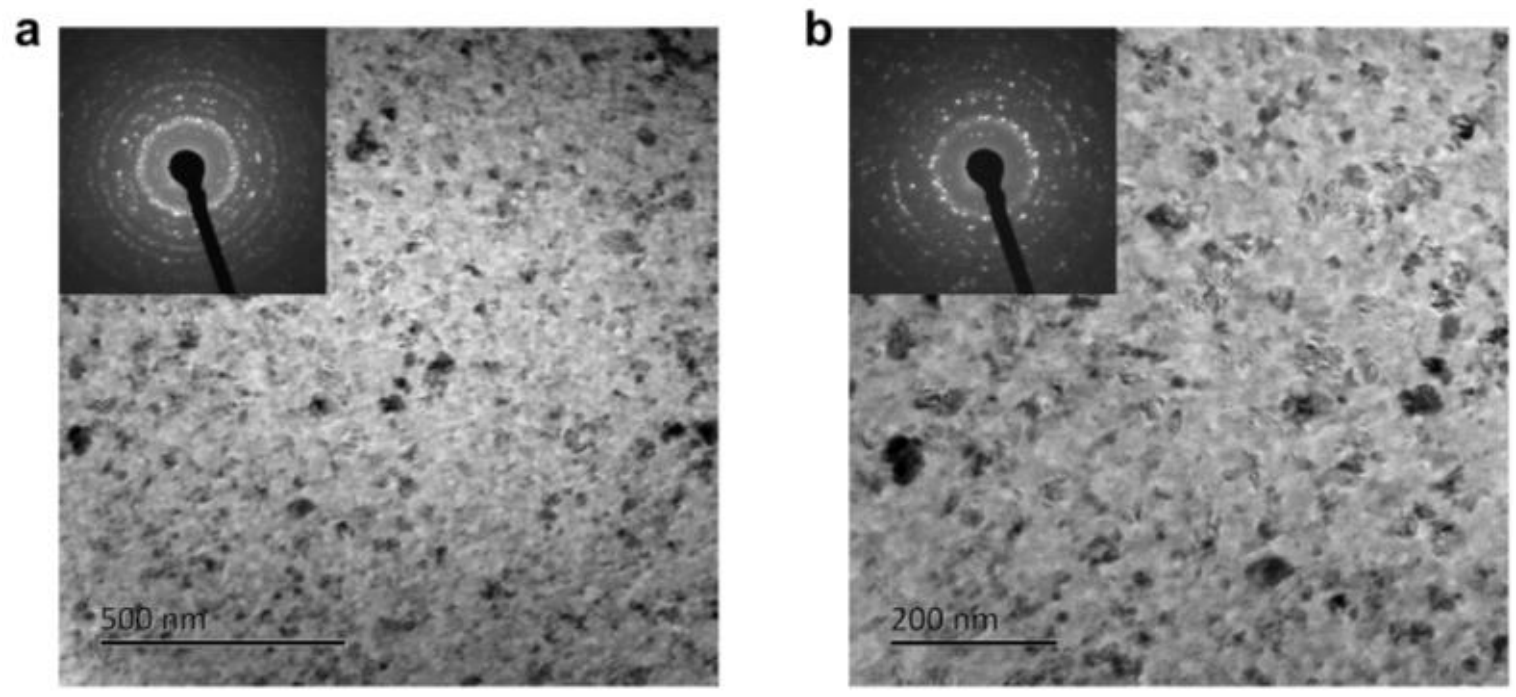

Figure R2-3 Microstructure and crystallinity of the fully crystallized material. a, Bright-field TEM image showing the overall morphology (scale bar: 500 nm). **b,** Higher-magnification TEM image revealing finer structural details (scale bar: 200 nm). The selected area electron diffraction (SAED) pattern (inset) exhibits sharp reflections from three distinct phases with no diffuse halos characteristic of an amorphous phase, confirming complete crystallization.

As noted in a recent review on crystallization of glass materials into transparent optical ceramics, to which our group contributed (Milisavljevic, I. et al., *International Materials Reviews*, 68, 648–676, 2023), a fully crystallized glass material is considered a ceramic when available characterization techniques (e.g., powder diffraction, electron microscopy) do not detect a residual glass phase. Conversely, any detectable glass phase would classify the material as a glass-ceramic. Based on this criterion, the combination of our XRD data, density measurements, and the newly obtained electron diffraction patterns provides a robust and convincing basis for concluding that our material is fully crystallized, rather than a glass-ceramic containing residual amorphous phases.

Changes: We have provided a more specific description of complete crystallization in the revised manuscript, supplemented by wide-area SAED patterns (Supplementary Fig. 2).

Comment 4: *Page 9, Line 205: The STEM-HAADF image in Figure 2 shows a sample with extremely fine and complex crystallization. Although the sample crystallized at 1100C for 2 hours is undoubtedly composed of very fine crystals, the grain size of 16 nm (mentioned in the conclusion) is questionable. Figures 2(b) and (c) show La(TiAl)O phases several tens of nanometers in size.*

Response: We thank the reviewer for the careful observation and this important question. The concern regarding the seemingly larger grains in Figures 2b and 2c is valid and provides an opportunity to clarify our statistical approach to grain size determination. The reported average grain size of ~16 nm in our conclusion is not a visual estimate from individual micrographs but is derived from systematic quantitative analysis, supported by the following:

1. Statistical Measurement from TEM: The grain size was systematically quantified using the intercept method on multiple representative TEM images. The grain size distribution histogram inserted in Figure 2a, for instance, is the result of statistical analysis over 424 measurement points, yielding an average size of approximately 16 nm.

2. Corroboration by XRD Refinement: The grain size estimated from X-ray diffraction line broadening using the Scherrer equation agrees well with the TEM statistical analysis, further confirming the nanocrystalline nature of the material.

3. Context for the Presented Micrographs: The specific fields of view shown in Figures 2b and 2c were selected primarily to clearly illustrate the three-phase coexistence and grain boundary interfaces. For this purpose, we chose regions with relatively distinct contrast and slightly larger grains (e.g., the ~20 nm $La(Al_{1/3}Ti_{2/3})O_3$ phase grain in Fig. 2c) to enhance feature visibility. These regions are not representative of the predominant grain size. To avoid any misinterpretation, we have added the following clarifying statement in the revised manuscript before the relevant figure

description: “To better visualize the three-phase microstructure, regions with distinct phase contrast and slightly coarser grains were selected for imaging.”

Therefore, the average grain size cited in our conclusion is a robust statistic validated by multiple methods. To further enhance precision, we have revised the grain size description in the manuscript from “~16 nm” to “<20 nm”, which more accurately reflects the size distribution while remaining consistent with the statistical results.

Changes: We have clarified how the average grain sizes were calculated when first reporting the data in the revised manuscript.

We would like to express our gratitude once again for your time and effort in reviewing our manuscript.

Reviewer #3

Opening comments: *In this work, the authors developed a novel material with high mechanical properties, a high refractive index, but a low vd, showing high potential in future optical devices. The manuscript is well - organized, but some comments still have to be made.*

Response: We sincerely thank reviewer #3 for the encouraging feedback and valuable suggestions. In response to the comments, we have substantially revised the manuscript. Specifically, we have provided more concrete evidence and discussion for complete crystallization, clarified the crystallization mechanism through the multi-phase interface locking strategy, and improved the presentation of optical and mechanical properties with updated figures and comparisons. We believe these revisions have further strengthened the clarity and impact of the work.

Comment 1: *In Fig. 1g, the sharp XRD peaks are not solid evidence for "complete crystallization". Since the authors use the glass crystallization method to prepare transparent ceramics, it is very important to confirm the full crystallization of the glasses. If not, it is just a common method to make transparent glass - ceramic, and nano - sized crystals in the glassy phase have been widely reported. In addition, some glass - ceramics with high crystallinity, even over 90%, have also been reported.*

Response: We thank the reviewer for raising this important point. We fully agree that sharp XRD peaks alone are insufficient to conclusively demonstrate "complete crystallization". Moreover, the Rietveld refinement results, which show a phase ratio of $La(Al_{1/3}Ti_{2/3})O_3$ to $Y_2(Zr_{0.6}Ti_{0.4})_2O_7$ of 85.3% to 14.7%, closely matching the nominal composition ratio of 88.6% to 11.4%, suggest that the vast majority of La_2O_3, Y_2O_3, TiO_2, and ZrO_2 present in the glass are consumed to form $La(Al_{1/3}Ti_{2/3})O_3$ and $Y_2(Zr_{0.6}Ti_{0.4})_2O_7$ nanocrystals.

To address this concern, we have provided additional evidence in the revised manuscript:

1. Density evolution as indirect evidence: In addition to XRD analysis, we measured the density of samples crystallized at different temperatures and for different durations. As shown in Fig. 1b, the density stabilized at 5.139 g cm^{-3} after crystallization at 1100 °C for 2 hours. This represents an increase of 12.57% relative to the glass precursor (4.565 g cm^{-3}), 2.7% relative to the sample crystallized at 900 °C for 2 hours (5.004 g cm^{-3}), and only about 0.14% difference relative to the samples crystallized at 1150 °C (5.132 g cm^{-3}) and 1200 °C (5.137 g cm^{-3}) for 2 hours, with no significant change (within 0.07%) observed upon further extending the holding time (5.135–5.142 g cm^{-3}). Based on the nominal composition, the theoretical density of the ceramic was calculated to be 5.142 g cm^{-3}, which is in good agreement with the measured density of 5.139 g cm^{-3}. This evolution in density, together with the consistency between the measured and theoretical values, provides further evidence that the crystallization process has reached completion.

2. Direct microstructural evidence via TEM electron diffraction: Following the reviewer's suggestion, we have now supplemented the manuscript with selected area electron diffraction (SAED) patterns acquired from wide regions of the sample (Supplementary Fig. 2; Figure R3-1 in this response). These patterns exhibit sharp diffraction spots with no detectable diffuse halos characteristic of an amorphous phase, providing direct evidence for the absence of a residual glassy phase. As noted in a recent review on crystallization of glass materials into transparent optical ceramics, to which our group contributed (Milisavljevic, I. et al., *International Materials Reviews*, 68, 648–676, 2023), a fully crystallized glass material is considered a ceramic when available characterization techniques (e.g., powder diffraction, electron microscopy) do not detect a residual glass phase (i.e., below the detection limit). Conversely, any detectable glass phase would classify the material as a glass-ceramic. Based on this criterion, the combination of our XRD data, density measurements, and the newly obtained electron diffraction patterns provides a robust and convincing basis for concluding that our material is fully crystallized, rather than a glass-ceramic containing residual amorphous phases.

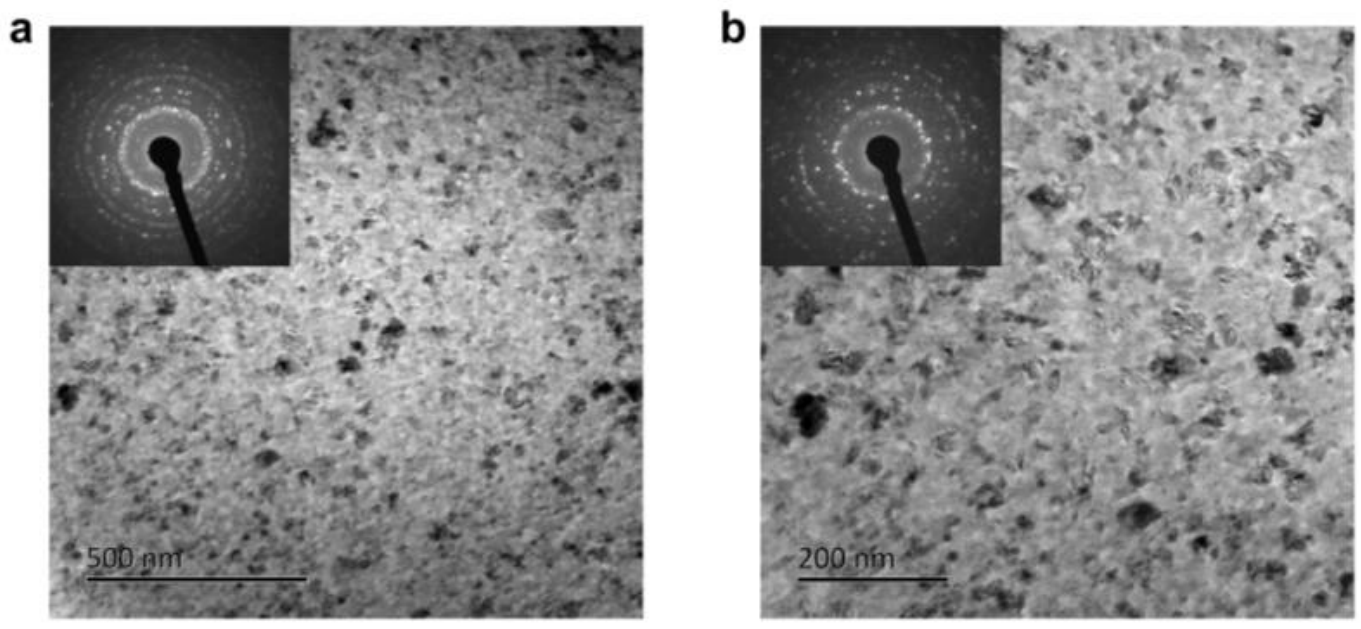

Figure R3-1 Microstructure and crystallinity of the fully crystallized material. a, Bright-field TEM image showing the overall morphology (scale bar: 500 nm). **b,** Higher-magnification TEM image revealing finer structural details (scale bar: 200 nm). The selected area electron diffraction (SAED) pattern (inset) exhibits sharp reflections from three distinct phases with no diffuse halos characteristic of an amorphous phase, confirming complete crystallization.

Changes: We have provided a more specific description of complete crystallization in the revised manuscript, supplemented by wide-area SAED patterns (Supplementary Fig. 2).

Comment 2: *It is suggested to add the scale bar for the samples in Fig 3a. And, usually the transmittance at 550 nm is used to evaluated for the transparency in the visible light range.*

Response: We thank the reviewer for these constructive suggestions. In response, we have revised the manuscript as follows: Regarding Figure 3(a), the sample photograph has been re-taken (as also noted in our response to Reviewer #2), and a scale bar has been added to the revised figure.

We also agree that transmittance at 550 nm is a standard metric for evaluating transparency in the visible range. Therefore, the transmittance value at this wavelength is now explicitly indicated in Fig. 3(b), and corresponding analysis and discussion have been added to the manuscript.

Changes: We have added the above analysis and discussions in the updated manuscript.

Comment 3: *The test method is significantly affect the results for the mechanical properties. In this work, the hardness was tested under a load of 4.9 N for 10 s. The applied load is smaller than the commonly used value. I wonder if it would affect the final results? In addition, it is suggestd to compare the modulus and KIC of nano-ceramics in this work to the reported transparent ceramics measured with the same method.*

Response: We thank the reviewer for the insightful questions regarding the mechanical property characterization. Regarding the load dependence of the measured fracture toughness, we have supplemented the Methods section with a detailed description and theoretical basis of the IF (Indentation Fracture) method. According to the fundamental principles of indentation fracture toughness evaluation, when appropriate empirical formulas are applied, the calculated K_{Ic}^{indent} values should theoretically be independent of the applied load. However, the choice of load is critical to ensure the development of a well-defined crack system. For reliable fracture toughness evaluation, the load must be sufficiently high to produce a c/a ratio >2.5 (where c is the distance from the indentation center to the crack tip). This indicates the formation of half-penny median-radial cracks, whereas when c/a < 2.5, the cracks extending from opposite indentation corners do not connect to each other (Palmqvist cracks) (Niihara, K. et al., *J Mater Sci Lett 1*, 13–16, 1982; Sellappan, P. et al., *Acta Materialia*, 61, 5949–5965, 2013). Particularly for finer-grained samples, relatively low loads are sufficient to generate half-penny median-radial cracks (Miyazaki, H. et al., *Ceramics International*, 36, 173–179, 2010). Conversely, excessively high loads may induce flow densification and inhibit crack propagation, leading to misestimated K_{Ic}^{indent} values (Dériano, S. et al., *Journal of Non-Crystalline Solids*, 344, 44–50, 2004).

For our material system, we selected 4.9 N as the optimal load after confirming that it yields a c/a ratio >2.5 without inducing severe ring cracks. When tested at a higher load of 9.8 N, the sample crystallized at 1100 °C for 2 h became prone to surface ring crack formation (Supplementary Fig. 5; Figure R3-2 in this response). Importantly, although the K_{Ic}^{indent} values obtained from the limited number of ring-crack-free indentations at 9.8 N were comparable to those at 4.9 N (Supplementary Fig. 5), the frequent occurrence of ring cracks at 9.8 N renders this higher load unsuitable for routine testing. The consistency and reliability of measurements at 4.9 N, combined with the absence of ring cracks, confirm its appropriateness for this material system.

Furthermore, following the reviewer's valuable suggestion, we have incorporated in the revised manuscript a comprehensive comparative analysis with other transparent ceramics reported in the references that were characterized using the same method. These data are summarized in Supplementary Table 4 (Table R3-1 in this response).

For hardness and Young's modulus, we added: "Comparative analysis with reported transparent ceramics (**Supplementary Table 4**) shows that the achieved hardness ranks among the higher values, surpassing those of $MgAl_2O_4$[60] (~16 GPa), $ZnGa_2O_4$[61] (10.56 ± 0.08 GPa), and Y_2O_3[62] (8.8 ± 0.2 GPa). The Young's modulus (270.7 GPa) is comparable to that of MgAlON[63] (288 GPa) and exceeds most reported values for transparent ceramics (**Supplementary Table 4**)."

For fracture toughness, we added: "As shown in **Fig. 4d**, crystallization at 1100 °C for 2 h increases K_{Ic}^{indent} by 55.4% (from 1.21 to 1.88 $MPa{\cdot}m^{1/2}$). This value substantially exceeds those of most oxide glasses reported to date (typically below 1 $MPa{\cdot}m^{1/2}$)[27] and approaches that of the LYTZA glass paracrystallized under high-pressure (15 GPa, 1000 °C) toughening (1.95 $MPa{\cdot}m^{1/2}$)[64]. It is also comparable to that of $ZnGa_2O_4$[61] transparent ceramics (1.83 $MPa{\cdot}m^{1/2}$) and falls within the range of fracture toughness values reported for most transparent ceramics (0.65–4.45 $MPa{\cdot}m^{1/2}$, **Supplementary Table 4**)."

These additions provide readers with a comprehensive assessment of the relative mechanical performance of our materials while maintaining appropriate context within the broader literature. We believe these revisions address both of the reviewer's concerns.

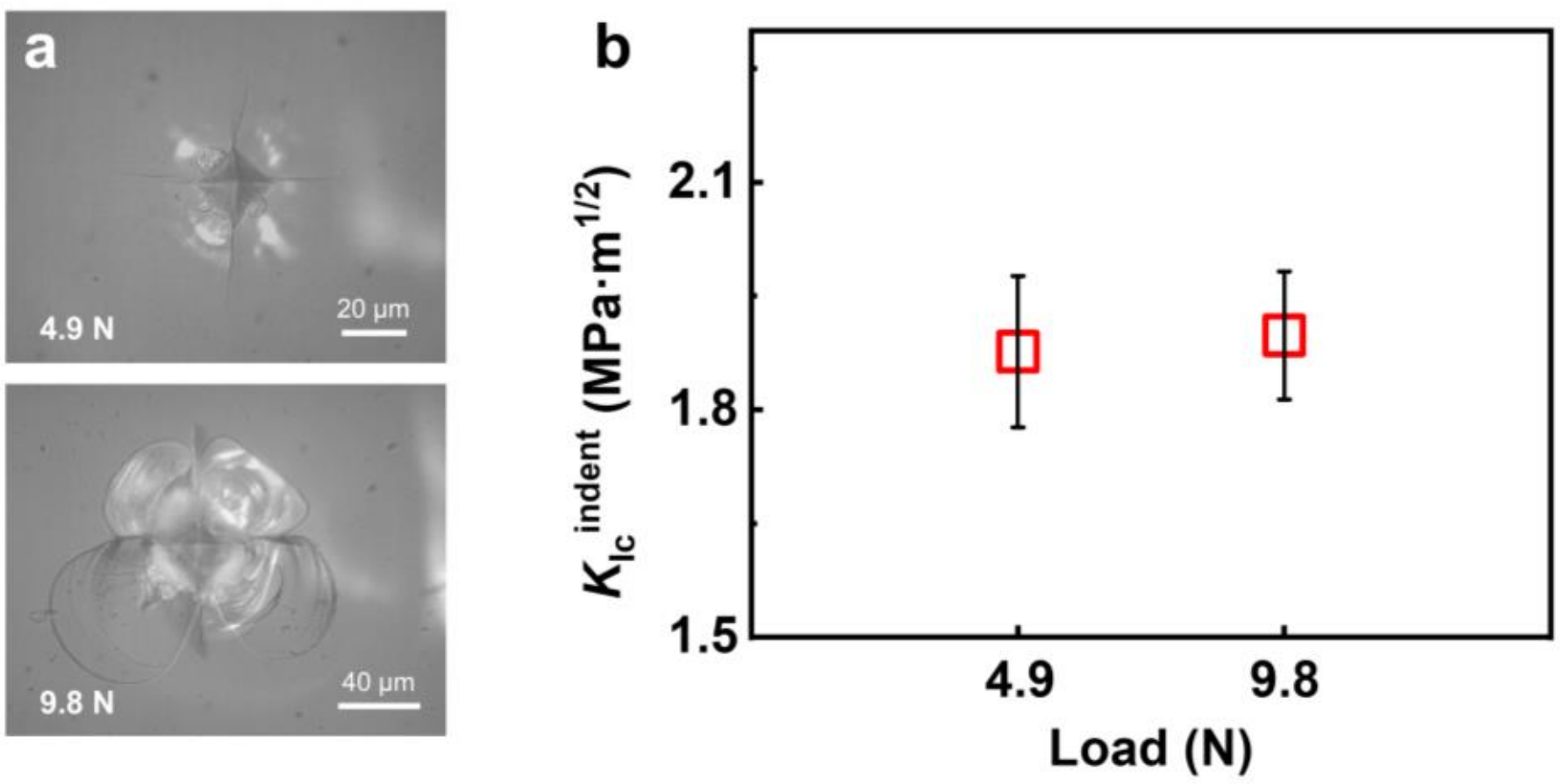

Figure R3-2 Load dependence of indentation fracture behavior. a, Representative optical micrographs of indentations on the sample crystallized at 1100 °C for 2 h, performed at 4.9 N and 9.8 N. At the higher load of 9.8 N, the sample became prone to surface ring crack formation. **b,** Indentation fracture toughness (K_{Ic}^{indent}) of the sample crystallized at 1100 °C for 2 h, measured under loads of 4.9 N and 9.8 N.

Table R3-1 Mechanical properties of transparent ceramics with different material systems: hardness, Young's modulus, and indentation fracture toughness.

**Material system**	**Hardness (GPa)**	**Young's modulus (GPa)**	**Indentation fracture toughness ($MPa\cdot m^{1/2}$)**
$MgAl_2O_4$ refs.60	16	210 ± 10	1.8 ± 0.1
$Mg_{0.27}Al_{2.58}O_{3.73}N_{0.27}$ (MgAlON) refs.63	15.1	288	-
$Y_3Al_5O_{12}$[1]	12.5	221	2.21
$Y_3Al_5O_{12}$[2]	22	271	2.23
$Y_3Al_5O_{12}$[2]	15	212	4.45
AlON[3]	15.6-16.6	-	1.6-2.0
0.1 at% Cr:Y_2O_3[4]	10.10 ± 0.04	-	1.11 ± 0.05
$ZnGa_2O_4$ refs.61	10.56 ± 0.08	227.04	1.83 ± 0.08
CaF_2:Yb[5]	3.2 ± 0.07	-	0.65 ± 0.12
8YSZ[6]	12.9±0.1	-	1.30±0.02
$Ca_3Al_2Si_3O_{12}$ refs.64 (paracrystallization)	7.7 ± 0.09	124.5 ± 1.4	1.99±0.06
$18.77La_2O_3–4.83Y_2O_3–28.22TiO_2–8.75ZrO_2–39.43Al_2O_3$ refs.64 (paracrystallization)	9.5±0.2	168.5±1.9	1.95±0.08
$LaAlO_3/tZrO_2$[7] (95.2% crystallinity)	19.05	-	2.64
$Sr_3Al_2O_6$ refs.41	10.5	150	-
$Y_3Al_5O_{12}–Al_2O_3$ refs.19	23.6	281	-
$GdAlO_3–Al_2O_3$ refs.44	22	284.7	-
This work: $La(Al_{1/3}Ti_{2/3})O_3–Y_2(Zr_{0.6}Ti_{0.4})_2O_7–Al_2O_3$	18.4	270.7	1.88

Changes: We have included in the Supplementary Information the fracture toughness obtained at higher loads, while a comparative analysis of hardness, Young's modulus, and fracture toughness with reference data has been added to the revised manuscript (Supplementary Table 4).

Comment 4: *Please clarify the crystallization and growth mechanism of the obtained nano-ceramics to highlight the novelty of this work. Regard to the title of this manuscript, glass-crystallized with nanosized crystals and ultra-fine size distribution is not limited. And how the multi-phase interface locking favor to obtain transparent nanocermaics?*

Response: We thank the reviewer for these insightful questions, which help us clarify the core

novelty of our work. Below we shall address the issues one-by-one.

1. On crystallization and growth mechanism

In conventional glass crystallization, single-phase nanocrystals nucleate and grow within a glass matrix, with grain growth typically controlled by short-range atomic rearrangement at the crystal–glass interface. This process often leads to rapid grain coarsening due to the large crystallization driving force. In contrast, the multi-phase interface locking mechanism demonstrated in this work fundamentally alters the growth kinetics. The simultaneous precipitation of three distinct crystalline phases from the homogeneous LYTZA glass creates a dense network of heterophase boundaries. For grain growth to proceed in such a multi-phase system, it requires not only the migration of these heterophase boundaries but also long-range atomic diffusion, where atoms must travel from source regions to sink regions of the same phase across distances that span multiple grains of different compositions. This coupling of grain growth to slower, compositionally constrained diffusion processes effectively suppresses coarsening, enabling the retention of nanocrystalline grains (<20 nm) even after complete crystallization.

It is instructive to compare this with our previous work on two-phase YAG-Al_2O_3 nanoceramics (Li, J. et al. *Nature Communications*, 9, 1175, 2018). In that system, Al_2O_3 acted as a physical barrier that imposed self-limited grain growth, achieving a minimum grain size of ~30 nm. While effective, the mechanism relied primarily on the passive presence of a second phase to obstruct coarsening. In contrast, the three-phase system developed here operates through a more fundamental kinetic constraint, grain growth is coupled to long-range, phase-specific diffusion, enabling the grain size to be pushed below 20 nm.

2. On the role of multi-phase interface locking in achieving transparency

While multi-phase coexistence typically degrades transparency due to scattering at phase boundaries, the nanocrystalline grain size achieved via interface locking fundamentally changes this paradigm. At grain sizes below 20 nm, far smaller than the wavelength of visible light (approximately 1/30 of green light wavelength), the phase boundaries themselves become sub-wavelength scattering centers. Under these conditions, Rayleigh scattering theory predicts that scattering losses scale with the sixth power of the grain size; thus, reducing grain size from, e.g., 100 nm to 20 nm reduces scattering by a factor of ~15,000.

In our material, the multi-phase interface locking mechanism enables this critical size threshold to be reached. The resulting transparency, maintained at appreciable levels (~70% in the visible range) despite complex three-phase coexistence, is therefore a direct consequence of the suppressed grain growth kinetics described above. This distinguishes our work from conventional glass-ceramics, where residual glassy phases or coarser grains often lead to significant scattering losses, and also

from our previous two-phase system, where the grain size remained above 30 nm. The key advance, therefore, lies in demonstrating that high transparency can be realized in a fully crystallized, multiphase ceramic when grain sizes are sufficiently refined—a concept validated by the interface locking mechanism.

We believe these clarifications highlight the novelty of our approach: not merely achieving nanocrystals via glass crystallization, but using multi-phase interface locking to kinetically arrest grain growth at a scale where optical scattering becomes negligible, thereby unlocking transparency in intrinsically scattering multi-phase systems.

Changes: We have incorporated the above points into the revised Discussion section to more clearly articulate the conceptual advance.

We would like to express our gratitude once again for your time and effort in reviewing our manuscript.

Reviewer #4

Opening comments: *In this manuscript, the authors report the fabrication of highly transparent multiphase nanoceramics derived from a high-entropy glass precursor, achieving an exceptionally small average grain size (~16 nm) despite the coexistence of three crystalline phases. By combining detailed structural characterization with optical and mechanical measurements, the authors claim that entropy-assisted glass crystallization suppresses grain growth and enables an unusual combination of multiphase coexistence and high transparency. The experimental work is carefully conducted, and the dataset is extensive. The reported grain size is indeed among the smallest achieved for bulk transparent ceramics prepared via glass crystallization. However, while the technical achievement is impressive, the manuscript in its current form lacks a sufficiently clear conceptual advance to fully justify publication in Nature Communications. Several key claims—particularly regarding the role of entropy—require clearer justification and sharper framing.*

Response: We sincerely thank the reviewer for the thoughtful and constructive feedback. We agree that a sharper conceptual narrative is needed. Following the suggestion, we acknowledge that the term "entropy" might have been overly emphasized as an explanatory mechanism in the current version. In the revised manuscript, we reframe the narrative to more precisely highlight the multi-phase interface locking effect as the central, direct mechanism enabling the key outcomes. We position the high-entropy glass precursor design primarily as the enabling condition that facilitates the simultaneous formation of multiple nanoscale phases, rather than as the primary explanatory driver.

Our revised discussion establishes a clearer logic: the multi-component nature of the glass precursor enables rapid multiphase nucleation, which in turn creates a dense network of heterophase boundaries. Once this nanostructure is formed, grain growth becomes kinetically constrained by long-range, phase-specific diffusion—a process fundamentally slower than the local atomic rearrangements governing grain growth in single-phase materials. This sluggish kinetics effectively suppresses coarsening, locking in the nanocrystalline grain size that directly enables the observed transparency and mechanical properties.

This reframing sharpens the manuscript's conceptual advance by highlighting a generalizable materials design principle: exploiting the inherently slow growth kinetics of multiphase nanocrystalline systems to achieve stable nanocrystalline microstructures with novel combinations of properties. We believe this clearer articulation strengthens the significance of our work. Thank you again for the insightful comments that have guided this important clarification.

Comment 1: *Insufficient clarity of the central conceptual advance: At present, the manuscript presents multiple strong results (ultrafine grain size, multiphase coexistence, high transparency,*

improved mechanical properties), but these are treated largely on an equal footing. As a consequence, the core message of the paper is diluted.

For a broad-scope journal such as Nature Communications, the manuscript must articulate a single, clearly defined conceptual advance. It is currently unclear whether the main contribution is:

- *the achievement of a record-small grain size via glass crystallization,*
- *the demonstration of high optical transparency in a multiphase ceramic,*
- *or the proposal of entropy as a governing parameter for crystallization kinetics.*

The authors are strongly encouraged to reformulate the manuscript around one dominant claim, with the remaining results positioned as supporting evidence rather than parallel achievements.

Response: We thank the reviewer for this insightful and constructive feedback. We fully agree that the manuscript's impact was diluted by treating multiple strong results on an equal footing, and we appreciate the guidance in sharpening our central conceptual advance.

Following the reviewer's suggestion, we have reformulated the manuscript around a single dominant claim: the achievement of a record-small grain size (<20 nm) in transparent ceramics via glass crystallization, enabled by multi-phase interface locking. This represents the core conceptual advance of our work, with the other findings, including high optical transparency and enhanced mechanical properties, positioned as direct consequences of this nanocrystalline microstructure rather than as parallel achievements. The proposal of entropy as a governing parameter for crystallization kinetics has been weakened.

To reflect this refocused narrative, we have reorganized the Results section into three logically connected subsections that establish: (1) how crystallization of the multi-component glass precursor yields three simultaneously precipitated phases; (2) direct microstructural evidence confirming the sub-20 nm grain size and how multi-phase interface locking enables this achievement; and (3) what this nanocrystalline microstructure enables in terms of optical transparency and enhanced mechanical properties.

This restructuring ensures that all presented results serve as supporting evidence for the central claim rather than as standalone achievements. Peripheral data have been relocated to the Supplementary Information to maintain focus on this central narrative. We believe this refocused narrative more effectively articulates the conceptual advance and trust that these revisions address the reviewer's concern. We are grateful for the reviewer's guidance in strengthening our manuscript.

Changes: We have revised the manuscript with better focus and weakened the discussion on entropy.

Comment 2: *Ambiguity in the role of entropy as a controlling parameter*

The manuscript repeatedly emphasizes the importance of configurational entropy in suppressing grain growth. However, the presented evidence does not yet convincingly demonstrate that entropy is the primary controlling factor, rather than a correlated descriptor.
A critical reader may reasonably ask:
Is entropy itself responsible for the suppressed grain growth, or are kinetic factors (e.g., reduced cation mobility, multiphase boundary pinning) the dominant mechanism?
To address this concern, the authors should consider one or more of the following:

- *Comparative experiments using lower-entropy compositions with otherwise similar chemistry,*
- *A more explicit kinetic or thermodynamic framework explaining how entropy quantitatively affects crystallization and grain growth,*
- *Or a reframing of entropy as a contributing, rather than governing, factor.*

Without such clarification, the entropy-based interpretation risks appearing post hoc.

Response: We thank the reviewer for this critical and insightful comment. We agree that the role of entropy must be clearly distinguished from the dominant kinetic mechanisms. In response, we have reframed the narrative throughout the revised manuscript to address this concern.

Specifically, we now note that while the multi-component glass precursor exhibits high configurational entropy, this feature is not invoked as a direct controlling factor for grain growth suppression. Instead, the key enabling characteristics of the precursor are simply its multi-component nature: it creates a chemically complex environment that slows cation diffusion required for phase partitioning, and it provides abundant nucleation sites that facilitate the simultaneous precipitation of multiple crystalline phases. These effects are necessary prerequisites for forming the dense heterophase boundary network, but they do not themselves constitute the primary growth inhibition mechanism. Once this multiphase nanostructure is established, grain growth becomes kinetically constrained by interface-dominated mechanisms: grain boundaries must migrate while atoms undergo long-range, phase-specific diffusion across dissimilar phases—a process fundamentally slower than the local atomic rearrangements governing grain growth in single-phase systems. This sluggish kinetics, rather than entropy, is what suppresses coarsening and locks in the nanocrystalline grain size.

Thus, our revised framework establishes a clear causal hierarchy in which the multi-component nature enables multiphase nucleation and slows initial diffusion, while kinetic factors (interface pinning and diffusion coupling) directly suppress grain growth, ultimately yielding nanocrystalline grains that enable the observed high transparency and enhanced mechanical properties. We believe this reframing addresses the reviewer's concern by explicitly separating the compositional complexity from the kinetic mechanisms that actively govern grain growth, avoiding any post hoc interpretation.

Changes: We have reorganized the logical flow of the manuscript, downplayed the role of entropy, and placed the main focus on the grain-growth suppression effect of multi-phase interface locking.

Comment 3: *Contextualization of the reported grain size*

The reported average grain size of ~16 nm is clearly remarkable. However, its significance is not sufficiently conveyed to non-specialist readers.

The authors should provide a systematic comparison with prior reports on transparent ceramics derived from glass crystallization, ideally in the form of a table or summary figure. This comparison should include:

- *Material systems,*
- *Processing routes,*
- *Achieved grain sizes,*
- *Optical transparency (where available).*

Such contextualization would substantially strengthen the manuscript and allow editors and readers to immediately appreciate the advance.

Response: We thank the reviewer for this valuable suggestion. We agree that systematic comparison with prior reports will help readers appreciate the significance of the achieved grain size.

To provide this context, we have compiled a comprehensive table (Table R4-1) summarizing previously reported transparent ceramics derived from glass crystallization. The table includes for each system: material system, processing route, grain size and optical property (transmittance and refractive index). This comparison clearly demonstrates that the <20 nm grain size achieved in our work, to the best of our knowledge, represents the finest grain size reported to date for fully crystallized transparent ceramics obtained via glass crystallization. The table has been placed in the "Results" section to provide readers with immediate context for evaluating the advance reported in this work. We believe this addition substantially strengthens the manuscript by contextualizing our findings within the broader literature.

Table R4-1 Comparison of transparent ceramics derived from glass crystallization: material systems, processing routes, achieved grain sizes, and optical transmittance.

Material system	Processing route	Grain size	Optical property	
			Transmittance	Refractive index
$Bi_{0.8}Nb_{0.8}Te_{2.4}O_8$[26]	510 °C, 1.5 h	8 μm	74% (3–5 μm)	2.24 ± 0.05 (1064 nm)
$Sr_{1+x/2}Al_{2+x}Si_{2-x}O_8$ ($0<x\leqslant 0.4$)[25]	1020 °C, 3 h	5–20 μm	T_{max}~90% (Vis–NIR)	1.59
α-$BaAl_4O_7$/β-$BaAl_4O_7$[16]	1000 °C, 2 h/ 1100 °C, 24 h	0.5–2 μm/ 1–5 μm	59.2%/39.5% (633 nm)	1.649/1.623 (514.5 nm)

β-$BaAl_4O_7$–$BaAl_2O_4$[22]	1100 °C, 20 h	1.4–3.2 μm	40–60% (1200 nm)	-
$Sr_3Al_2O_6$[41]	840 °C, 5 h	0.5–2 μm	T_{max}~87.4±0.5% (300 nm–6 μm)	1.70 ± 0.02
$Y_2CaAl_4SiO_{12}$[42]	1050 °C, 10 h/ 1150 °C, 10 h/ 1250 °C, 10 h	21.4 ± 6.9 nm/ 150.8 ± 51.3 nm/ 760 ± 250 nm	28% (450 nm, 1250 °C)	-
$Ca_3Al_2Si_3O_{12}$[9]	>10 GPa, 1400 °C, 2 h	30–50 nm	T_{max}~86.7% (400–800 nm)	-
$LaAlO_3$/t-ZrO_2[43]	1200 °C, 2 h	40 nm (95.2% crystallinity)	55% (800 nm)	-
$Lu_3Al_5O_{12}$–Al_2O_3[20]	1100 °C, 2 h	65.7 ± 2.3 nm	82% (780 nm)	-
$GdAlO_3$–Al_2O_3[44]	1000 °C, 2 h	42 ± 2 nm	77% (780 nm)	-
$Y_3Al_5O_{12}$–Al_2O_3[19]	1100 °C, 2 h	30 nm	T_{max}~87% (NIR)	1.77 (589 nm)
This work: $La(Al_{1/3}Ti_{2/3})O_3$–$Y_2(Zr_{0.6}Ti_{0.4})_2O_7$–$Al_2O_3$	1100 °C, 2 h	16 nm	69.5% (700 nm)	2.01 (587.6 nm)

Changes: We have added to the revised manuscript a comprehensive comparison table summarizing previously reported transparent ceramics derived from glass crystallization, along with the corresponding discussion.

Comment 4: *Multiphase coexistence versus transparency: missed opportunity*

One of the most intriguing aspects of this work is the coexistence of three crystalline phases without significant loss of optical transparency. This result runs counter to conventional expectations in ceramic optics.

However, this apparent contradiction is not sufficiently emphasized or conceptually exploited. The authors should explicitly address why multiphase coexistence, typically detrimental to transparency, does not degrade optical performance in this case, and how the ultrafine grain size fundamentally alters this paradigm.

Framing this point more explicitly could significantly enhance the manuscript's conceptual impact.

Response: We thank the reviewer for this insightful observation. We agree that the coexistence of three crystalline phases without significant loss of transparency is one of the most intriguing and counterintuitive aspects of this work, and we appreciate the opportunity to develop this point more explicitly.

In the revised manuscript, we have substantially rewritten the optical properties section to frame this as a size-dominated transparency paradigm enabled by nanocrystallize grains (<20 nm). Specifically:

1. Explicit framing of the apparent contradiction: We now open the discussion by explicitly stating that multiphase coexistence without transparency loss runs counter to conventional expectations in ceramic optics, where each additional phase boundary is typically considered an

inevitable source of scattering. We position this seeming paradox as the central conceptual advance of our work.

2. Mechanistic explanation: We clearly articulate that the conventional penalty of multiphase scattering is not an inevitable rule of optics, but rather a consequence of micrometer-scale microstructures. When the grain size is forced below ~20 nm (approximately 1/30 of the wavelength of green light), the phase boundaries themselves become too small to interact with visible light as independent scatterers. Citing established scattering models (Apetz and van Bruggen; Harris et al.), we show that once grain size falls below λ/10, the scattering cross-section of a phase boundary scales with the sixth power of its size, becoming negligible regardless of the number of interfaces or their refractive index mismatch. Thus, multiple phases, though normally detrimental, become optically invisible when sufficiently refined.

3. Hierarchical attribution: We restructure the contributing factors to emphasize that while densification and cubic symmetry are important conditions, the nanocrystallize grain size (<20 nm) is the uniquely enabling factor that fundamentally alters the scattering paradigm. Without it, even a fully dense, optically isotropic multiphase ceramic would still scatter light at every phase boundary. The nanocrystallize grains effectively "hide" the multiphase interfaces from incident light, allowing the material to behave optically as a homogeneous medium despite its structural complexity.

4. Generalizable principle: We now present this as a generalizable design principle, optical transparency can be achieved in intentionally complex multiphase systems by reducing all microstructural features (including phase boundaries) well below the wavelength of light. This principle liberates transparent ceramic design from the traditional constraint of phase purity and opens new possibilities for materials with deliberately engineered multiphase architectures.

We believe this reframing transforms what was previously presented as a descriptive result into a conceptually impactful demonstration of how nanoscale size effects can overcome classical limitations in optical materials, turning a conventional weakness (multiphase coexistence) into an irrelevant feature. The revised manuscript now emphasizes this point throughout, ensuring readers appreciate both the counterintuitive nature of the result and its broader implications.

Changes: We have substantially rewritten the optical properties section to highlight the coexistence of three crystalline phases without significant loss of optical transparency.

Comment 5: *Overextension of the results: The manuscript covers a wide range of properties (structural, optical, mechanical, thermodynamic), which risks overextending the narrative. Some sections read as descriptive rather than hypothesis-driven. For improved focus, the authors should*

consider prioritizing the results most directly supporting the central claim, while relocating secondary data to the Supplementary Information.

Response: We thank the reviewer for this valuable suggestion. In response, and consistent with our revisions addressing Comment 1, we have streamlined the manuscript to focus on the results most directly supporting our central claim (the achievement of record-small grain size via glass crystallization). Peripheral data and discussions have been relocated to the Supplementary Information, and the narrative has been sharpened by substantially streamlining descriptive content to maintain focus on the nanocrystalline microstructure and its implications. These changes have significantly improved the focus and readability of the manuscript.

Minor Comments

1. The Introduction is overly long and at times resembles a review article. Streamlining would improve readability.

Response: We thank the reviewer for this valuable feedback. In response, we have thoroughly streamlined the Introduction by removing content only tangentially related to the core theme, thereby sharpening the focus on the key scientific challenge of grain size refinement in transparent ceramics. The retained content has also been refined for conciseness, eliminating redundant expressions to ensure a more compact and fluent narrative. We believe the revised Introduction now more effectively orients readers toward the central problem and novelty of our study.

Changes: We have shortened the introduction section.

2. Claims of "first" or "unprecedented" should be supported by explicit comparisons or softened where appropriate.

Response: We thank the reviewer for this important reminder regarding the use of absolute claims. To ensure rigor and avoid overstatement, we have carefully revised the manuscript by softening such language where appropriate (e.g., replacing "unprecedented" with more cautious phrasing). Importantly, these modifications do not diminish the significance of our findings—the systematic comparisons with prior work (as summarized in the newly added table addressing comment 3) clearly demonstrate that the <20 nm grain size achieved in this study represents a substantial advance over previously reported values. We believe this revised phrasing strikes an appropriate balance between accurately conveying the novelty of our results and maintaining scholarly caution.

Changes: We have softened the claims in the revised manuscript.

3. The limitations of Scherrer-based grain size estimation should be discussed more explicitly, particularly in comparison with STEM-derived values.

Response: We thank the reviewer for raising this important point. We agree that a transparent discussion of methodological considerations strengthens the credibility of the results.

In the revised manuscript, we have explicitly addressed the limitations of Scherrer-based grain size estimation. Specifically, we have added the following statement: "The Scherrer equation provides a convenient estimate of the coherent diffraction domain size, but it assumes spherical, strain-free crystallites and may be influenced by microstrain and instrumental broadening (Hargreaves, J. S. J., *Catal. Struct. React.* 2, 33–37, 2016), despite our correction for the latter. These values should therefore be interpreted as volume-weighted averages rather than absolute measures of physical grain dimensions (Drits, V., Środoń, *Clays Clay Miner.* 45, 461–475 1997)." We believe this balanced discussion provides readers with a clear understanding of how the grain size was determined and the confidence level of the reported values, while also contextualizing the close agreement between XRD-derived and STEM-measured grain sizes discussed in the main text.

Changes: We have added the discussion on the limitations of Scherrer-based grain size estimation in the revised manuscript.

4. Several causal statements rely on speculative phrasing (e.g., "may be attributed to"). Where possible, these should be strengthened or qualified more precisely.

Response: We thank the reviewer for this careful observation. We agree that precise causal language is essential for scientific rigor, and we have revised the manuscript accordingly.

Throughout the manuscript, where sufficient evidence supports a direct causal relationship, we have replaced tentative expressions (e.g., "..., the fracture toughness decreases to 1.71 MPa·$m^{1/2}$, which may be attributed to the excessive growth of grains.") with more definitive language (e.g., "This decline is associated with excessive grain growth and the development of heterogeneous residual stresses arising from mismatched thermal expansion coefficients among the different phases[60]"). For instances where some uncertainty remains appropriate, we have qualified the statements more precisely to accurately reflect the strength of the evidence.

These revisions have enhanced the clarity and authority of our mechanistic interpretations while maintaining scientific caution where warranted. We believe the revised manuscript now presents causal relationships with appropriate confidence levels throughout.

Changes: We have replaced speculative phrasing with more definitive language throughout the manuscript.

We would like to express our gratitude once again for your time and effort in reviewing our manuscript.

Reviewer #1

Opening comments: *All the concerns of this reviewer have been properly and adequately addressed, and the work may now be accepted for publication.*

Response: We sincerely thank the reviewer for the positive assessment and for confirming that all concerns have been adequately addressed. We are very grateful for the reviewer's time and constructive input throughout the review process.

Reviewer #2

Opening comments: *The authors have added substantial results and discussions, particularly regarding the evaluation of optical and mechanical properties.*

Response: We thank the reviewer for the encouraging feedback and for recognizing the substantial additions to the manuscript, particularly regarding the evaluation of optical and mechanical properties. We greatly appreciate the reviewer's valuable suggestions that helped improve our work.

Reviewer #4

Opening comments: *The revised manuscript has substantially improved in focus and now presents a clearer central message centered on multi-phase interface locking and sub-20 nm grain-size control. The role of entropy has been appropriately deemphasized, and the discussion of transparency in a multiphase nanoceramic is much clearer. I consider the revision largely responsive to my major concerns.*

Response: We are very grateful for the reviewer's positive assessment and constructive feedback throughout the revision process. We are pleased that the revised manuscript now better conveys the central message and addresses the major concerns. Thank you very much for your time and insightful guidance.

Before final acceptance, I recommend minor textual revisions:

Comment 1: *(i) ensure consistency between the response letter and manuscript regarding the location of the comparison table; if the table is central to contextualizing the grain-size advance, it should preferably appear in the main text rather than only in the Supplementary Information;*

Response: We apologize for the inconsistency between the response letter and the manuscript regarding the location of the comparison table. We had carefully considered whether to place the table in the main text, but after thorough deliberation we decided to keep it in the Supplementary Information to maintain the focus and readability of the main text.

We fully agree with the reviewer that the table is important for contextualizing the grain-size advance. Therefore, while the detailed comparison remains in **Supplementary Table 3**, we have explicitly stated the key conclusion in the main text (Results section): the sub-20 nm grain size achieved in our work is remarkably fine, with a direct reference to **Supplementary Table 3**. We believe this arrangement still allows readers to immediately appreciate the advance while keeping the main text concise.

We hope this decision is acceptable to the reviewer. We remain open to further discussion and will adjust the table location if needed.

Changes: We have retained the comparison table as Supplementary Table 3.

Comment 2: *(ii) soften absolute wording such as "record-fine" unless fully supported by the main-text comparison;*

Response: We thank the reviewer for this valuable suggestion. We have softened the absolute

wording by replacing "record-fine" with "a fine grain size" in the manuscript. We have also carefully reviewed the entire text and confirmed that no other unsupported absolute claims (e.g., "unprecedented", "first") remain. All descriptive terms are appropriately supported by the data.

Changes: We have replaced "record-fine" with "a fine grain size" in the relevant sentence and removed any other unsupported absolute claims throughout the manuscript.

Comment 3: *(iii) moderate remaining strong causal language in the Abbe-number and mechanical-property discussions, where the mechanistic evidence is less direct than for grain-size suppression.*

Response: We thank the reviewer for this careful guidance. We have moderated the causal language in the Abbe-number and mechanical-property sections. For the Abbe-number discussion, we removed strong mechanistic claims and now simply describe the correlation between nanostructure and v_d changes. For mechanical properties, we replaced terms such as "governed by" and "confirms" with more cautious phrasing ("is associated with", "illustrates", "can facilitate", etc.). These revisions bring the language in line with the strength of the available evidence.

Changes: We have moderated causal language in the Abbe-number and mechanical-property sections. All affected sentences have been revised accordingly in the manuscript.

With these minor changes, I believe the manuscript would satisfactorily address my previous concerns.

We would like to express our gratitude once again for your time and effort in reviewing our manuscript.
